# VvSWEET7 Is a Mono- and Disaccharide Transporter Up-Regulated in Response to *Botrytis cinerea* Infection in Grape Berries

**DOI:** 10.3389/fpls.2019.01753

**Published:** 2020-01-27

**Authors:** Richard Breia, Artur Conde, Diana Pimentel, Carlos Conde, Ana Margarida Fortes, Antonio Granell, Hernâni Gerós

**Affiliations:** ^1^ Centre of Molecular and Environmental Biology (CBMA), Department of Biology, University of Minho, Braga, Portugal; ^2^ Centre for the Research and Technology of Agro-Environmental and Biological Sciences (CITAB), University of Trás-os-Montes e Alto Douro, Vila Real, Portugal; ^3^ University of Lisbon, Lisbon Science Faculty, BioISI, Campo Grande, Lisbon, Portugal; ^4^ i3S-Institute of Research and Innovation in Health, University of Porto, Porto, Portugal; ^5^ IBMC-Institute for Molecular and Cell Biology, University of Porto, Porto, Portugal; ^6^ Institute of Molecular and Cellular Biology of Plants, Spanish National Research Council (CSIC), Polytechnic University of Valencia, Valencia, Spain

**Keywords:** sugar transporter, SWEET, biotic stress, grey mold, *Botrytis cinerea*, plant pathogens, grape berry, grapevine

## Abstract

The newly-identified SWEETs are high-capacity, low-affinity sugar transporters with important roles in numerous physiological mechanisms where sugar efflux is critical. SWEETs are desirable targets for manipulation by pathogens and their expression may be transcriptionally reprogrammed during infection. So far, few plant SWEET transporters have been functionally characterized, especially in grapevine. In this study, in the *Botrytis*-susceptible variety “Trincadeira,” we thoroughly analyzed modifications in the gene expression profile of key *SWEET* genes in *Botrytis cinerea*-infected grape berries. VvSWEET7 and VvSWEET15 are likely to play an important role during fruit development and *Botrytis* infection as they are strongly expressed at the green and mature stage, respectively, and were clearly up-regulated in response to infection. Also, *B. cinerea* infection down-regulated *VvSWEET17a* expression at the green stage, *VvSWEET10* and *VvSWEET17d* expression at the veraison stage, and *VvSWEET11* expression at the mature stage. VvSWEET7 was functionally characterized by heterologous expression in *Saccharomyces cerevisiae* as a low-affinity, high-capacity glucose and sucrose transporter with a *K*_m_ of 15.42 mM for glucose and a *K*_m_ of 40.08 mM for sucrose. VvSWEET7-GFP and VvSWEET15-GFP fusion proteins were transiently expressed in *Nicotiana benthamiana* epidermal cells and confocal microscopy allowed to observe that both proteins clearly localize to the plasma membrane. In sum, VvSWEETs transporters are important players in sugar mobilization during grape berry development and their expression is transcriptionally reprogrammed in response to *Botrytis* infection.

## Introduction

Grapevine (*Vitis vinifera* L.) is prone to a wide range of pathogens that cause production and quality losses. Plant pathogens are separated in three broad classes according to their modes of nutrition. Biotrophs are pathogens that need a living host to survive, having developed strategies to obtain nutrients from the host without inducing plant defense mechanisms or cell death (Perfect et al., 1999; Glazebrook, 2005). For nutrient uptake and secretion of limited amounts of cell wall-degrading enzymes, they develop specialized structures, including haustoria (Mengiste, 2012). Necrotrophs obtain nutrients from dead cells, which are killed during the infection process. They cause necrosis secreting hydrolytic enzymes that degrade the cell wall (van Kan, 2006), secrete toxins (Govrin et al., 2006; Dalmais et al., 2011), and also hijack the plant enzymatic machinery, promoting programmed cell death (Cantu et al., 2009). The hemibiotrophs pathogens are able to switch lifestyles at specific developmental stages—they display an early biotrophic phase followed by a necrotrophic phase—or at different environmental conditions (Glazebrook, 2005; Kleemann et al., 2012). *Botrytis cinerea*, the causal agent of the grey mold disease in more than 200 plants (Elad et al., 2004), is one of the most important grapevine pathogens (Haile et al., 2017). It is a necrotrophic fungus with a short biotrophic phase (Veloso and van Kan, 2018). Usually, *B. cinerea* infection begins by airborne conidia that settle in the host superficial cells (Nair et al., 1995; Elmer and Michailides, 2004). In the vineyard, this pathogen is part of the microflora and infects primarily ripe berries; however, inoculation of *Botrytis* spores often occurs during the onset of the grape berry (McClellan and Hewitt 1973; Nair et al., 1995; Keller et al., 2003; Pezet et al., 2003). During development, grape berries suffer several modifications that decrease its natural resistance to the pathogen. The cuticle and the cell-wall suffer modifications that lower their mechanical resistance (promoting micro-fractures), the bunches become more compact, (Vail and Marois, 1991; Kretschmer et al., 2007), the sugar levels increase and the concentration of organic acids and several compounds related to biotic resistance decrease (Miedes and Lorences, 2007; Cantu et al., 2008; Cantu et al., 2009; Centeno et al., 2011; Blanco-Ulate et al., 2013; Prusky et al., 2013; Blanco-Ulate et al., 2015). During infection, *B. cinerea* secretes several compounds and enzymes to macerate and penetrate the host tissue (Dulermo et al., 2009). Additionally, the fungus can manipulate the host biological processes to its own gain, promoting the programmed cell death machinery (Veloso and van Kan, 2018) or hastening the ripening process. In infected immature grape berries, Agudelo-Romero et al. (2015) observed a large transcriptional activation of genes related to the maturation process and an accumulation of several compounds associated with maturation. A similar highjack of plant metabolism by pathogen agents was also observed in other plant species (Baker et al., 2012; Morkunas and Ratajczak, 2014). Different studies reported an increase of invertase activity in different plants in response to powdery mildew or other diseases (Ruiz and Ruffner, 2002; Roitsch et al., 2003; Kocal et al., 2008; Siemens et al., 2011). Also, up-regulation of sugar transporters was observed during pathogen infection in *Arabidopsis thaliana* and *Pinus pinaster* cultured cells (Truernit et al., 1996; Azevedo et al., 2006).

Recently identified and somewhat different from the common sugar transporting proteins, SWEETs transporters were characterized as bi-directional, low-affinity sugar carriers, probably operating by an uniport mechanism (Chen et al., 2010). Generally, in angiosperms, the SWEET family is composed of 20 members and have different physiological roles, typically related with sugar efflux mechanisms. In *Arabidopsis*, SWEET transporters are essential members in nectar secretion (Lin et al., 2014), phloem sugar loading (Chen et al., 2012), seed nutrient filling (Chen et al., 2015a), and pollen feeding and vacuolar fructose storage (Chardon et al., 2013; Klemens et al., 2013; Guo et al., 2014). In maize, ZmSWEET13a, b, and c are key components in apoplasmic phloem loading (Bezrutczyk et al., 2018); in rice, OzSWEET11 and 15 are crucial components in seed filling (Yang et al., 2018). Also, in *Arabidopsis*, AtSWEET13 and 14 are capable of transporting multiple forms of gibberellins, revealing an exciting plasticity of these transporters (Kanno et al., 2016). In the grapevine, the SWEET family is composed by 17 members, and only VvSWEET4 and VvSWEET10 were functionally characterized (Chong et al., 2014; Zhang et al., 2019).

Similar to their effect in other sugar transporters, pathogens are also able to alter the expression profile of different SWEET genes (reviewed by Chen et al., 2015b and Julius et al., 2017). In rice, some members of this family (*OsSWEET11*, *OsSWEET12*, and *OsSWEET14*) were up-regulated during *Xanthomonas oryzae* infection (Chu et al., 2006; Yang et al., 2006; Antony et al., 2010; Chen et al., 2010). In cassava (*Manihot esculenta*), *Arabidopsis*, citrus (*Citrus paradisi* and *Citrus sinensis*), barrel clover (*Medicago truncatula*), grapevine, and sweet potato (*Ipomoea batatas*), up-regulation of SWEET genes was also observed during pathogen attack (Chen et al., 2010; Chong et al., 2014; Cohn et al., 2014; Hu et al., 2014; Li et al., 2017). An increased number of sugar transporters can lead to sugar accumulation in the apoplast, which in turn is used as source of carbon and energy by the pathogen (Wright et al., 1995; Clark and Hall, 1998; Lemoine et al., 2013), but pathogens can also affect negatively the expression of SWEET genes. *Botrytis cinerea* infection in tomato cotyledons promoted a down-regulation of different members of the *SWEET* family (Asai et al., 2016). This form of regulation can lead to the disruption of sugar signaling pathways related to defense responses to biotic stress (Berger et al., 2007; Sade et al., 2013; Morkunas and Ratajczak, 2014).

Thus, considering the possible role of SWEET transporters either by accentuating or counteracting the infection, we aimed in this study to confirm the hypothesis that the infection by *B. cinerea* causes a transcriptional reprogramming of the expression of *SWEET* genes in grape berry tissues. Moreover, considering its naturally significant steady state transcript abundance and up-regulation by *B. cinerea* infection in berries, we functionally characterized VvSWEET7, unveiling its sub-cellular localization and sugar transport kinetics resorting to two heterologous expression models, tobacco and yeast, respectively.

## Materials and Methods

### Biological Material

Clusters of Trincadeira grapes were infected by inoculation with a conidial suspension of *B. cinerea* at EL29 (peppercorn-size, early green stage) in very well-established and standardized conditions according to Agudelo-Romero et al. (2015) and Coelho et al. (2019). Inoculation was performed at the same time in multiple clusters in very similar conditions, particularly in size, appearance, exposure to light, canopy densities, and plant orientation between them and also identical to control clusters. Samples were harvested at three developmental stages: at green (EL32), veraison (EL35), and mature (EL38) (Coombe, 1995). For each treatment (infected and control) and ripening stage, three biological replicates were collected at around 10 a.m., each one constituted by a composite pool of at least 12 berries collected from different clusters from three different plants. Every collected infected berry had the same infection appearance and visual symptoms that were in fact similar between all infected clusters, as the inoculation was performed at the same time in all berry clusters. Thus, both control and infected collected grape berries were well representative of their physiological condition. The collected samples were frozen in liquid nitrogen and stored at -80°C. Prior to RNA extraction, the seeds of each of the three sampled biological replicates were removed and the remaining tissues were ground in liquid nitrogen to a fine powder. For all analyses performed on grape berry tissues in this work, each of the three biological replicates was used for a different RNA extraction and independent qPCR analysis, with each qPCR analysis having three internal technical replicates.

Cell suspension cultures of *V. vinifera* L. (Cabernet Sauvignon Berry - CSB) were freshly established from somatic callus that had been previously initiated from Cabernet Sauvignon berry pulp at Serge Delrot’s lab (ISVV, Bordeaux) according to Calderón et al., 1994. They were maintained in 250 ml flasks at 25°C in the dark on a rotator shaker at 100 rpm on modified Murashige and Skoog (MS) medium (Murashige and Skoog, 1962; Decendit et al., 1996), supplemented with 2% (w/v) sucrose as carbon and energy source. The suspension-cultured cells were sub-cultured weekly by transferring 10 ml aliquots into 40 ml of fresh medium. *B. cinerea* growth and mycelia harvest were performed according to Azevedo et al. (2006). The fungus was cultivated in potato dextrose (PD) liquid medium at 25°C with agitation (150 rpm). Mycelia were harvested from 12-d cultures by centrifugation at 5,000×g for 5 min, followed by resuspension in sterile water. The mycelia were autoclaved and then lyophilized for 48 h and ground with a mortar and pestle to a fine powder. For elicitation experiments, grapevine suspension cells were harvested at the mid-exponential growth phase, centrifuged at 5.000 xg for 5 minutes, and resuspended in MS medium at a final density of 0.1 g F.W. mL^-1^. *Botrytis* mycelia extract was added to the suspension cell culture at a final concentration of 2 mg mL^-1^. Then, the control and elicited suspension cultures were incubated in the dark at 25°C on a rotatory shaker at 100 rpm. After 48 hours of incubation, cells were filtered through GF/C filters (Whatman) and samples were washed with deionized water and immediately frozen in liquid nitrogen and ground to a fine powder with a mortar and pestle. For both *Botrytis*-elicited and control suspension cell cultures, three biological replicates were used.

### RNA Extraction

An initial amount of 200 mg of ground control or infected grape berry tissues from each of the three biological replicates sampled was used for total RNA extractions, as well as pulverized *Botrytis*-elicited and control suspension cell culture samples, following the method described by Reid et al. (2006) combined with in-column purification using the RNeasy Plant Mini Kit (Qiagen). After isolation and verification of RNA purity, treatment with DNase I (Qiagen) was performed and cDNA was synthesized from 1 µg of total RNA using the Xpert cDNA Synthesis Master-mix Kit (GRISP).

### Gene Expression Analysis by qPCR

The expression profile of *VvSWEET* genes in all the studied samples (control and infected grape berries and control and *Botrytis*-elicited suspension cell cultures) was analyzed by real-time qPCR performed using cDNAs obtained from RNAs extracted from each of the three composite pools of grape berry samples that constitute the three biological replicates of each condition in this study. Real-time qPCR was performed with Xpert Fast SYBR Blue (GRISP) using 1 µL of diluted cDNA (1:10) in a total of 10 µL of reaction mixture per well. For reference genes, *VvACT1* (actin) and *VvGAPDH* (glyceraldehyde-3-phosphate dehydrogenase) were used, as they are considered extremely adequate reference genes for gene expression normalization purposes in qPCR analyses in grapevine (Reid et al., 2006). Specific primers used for each studied gene are listed in the **Supplementary Table 1**. Melting curve analysis was performed for specific gene amplification confirmation. Stability of the reference genes was confirmed by the automatic M-value analysis performed by the Bio-Rad^®^ CFX Manager 2.0 Software. For each gene, the relative gene expression values were obtained following calculation by the Bio-Rad^®^ CFX Manager 2.0 Software. For each of the three biological replicates, after RNA extractions and cDNA synthesis, an independent qPCR analysis was performed with three internal technical replicates.

### *VvSWEET7 *and *VvSWEET15* Molecular Cloning and Construction of Destination Plasmids

The putative sugar transporter genes, *VvSWEET7* and *VvSWEET15*, were cloned by Gateway^®^ technology. Primers pairs, designed with the attB sequences (**Supplementary Table 1**) for site-specific recombination with the entry plasmid pDONR221, were used for PCR amplification of the target genes. Subsequently, recombination of the attB-containing target genes with the entry plasmid was performed using the BP clonase enzyme. The target genes carried in the entry plasmid were then recombined by the LR clonase enzyme into the *pH7WGF2* plasmid (containing the *egfp* gene) for sub-cellular localization and into the *pYES-DEST52* plasmid for heterologous expression in yeast. All constructs were confirmed by sequencing.

### Sub-Cellular Co-Localization Studies in Tobacco Leaves

The N-terminally fused constructs *pH7WGF2-GFP-VvSWEET7* and *pH7WGF2-GFP-VvSWEET15* were introduced in *Agrobacterium tumefaciens* strain EHA105; transient transformation of tobacco leaves (*Nicotiana benthamiana*) was performed according to Sparkes et al. (2006). Transformed *Agrobacterium* cells were inoculated overnight in liquid LB medium with the appropriate antibiotic selection up to the exponential-stationary phase, and then diluted to OD_600nm_ = 0.1 with infiltration buffer (50 mM MES pH 5.6, 2 mM Na_3_PO_4_, 0.5% glucose, 100 µM acetosyringone). Cells were then incubated until the culture reached an OD_600nm_ = 0.2. Leaves of three different four-week-old tobacco plants were infiltrated with the *Agrobacterium* culture and, after 2 days, discs of the infected leaves were observed at the scanning confocal microscope (Leica TCS SP5IIE-Leica Microsystems). Data stacks were analyzed and projected using ImageJ 1.42m software (http://rsb.info.nih.gov/ij/). The plasma membrane marker used was the plasma membrane aquaporin AtPIP2;1 C-terminally fused to the fluorescent protein mCherry (AtPIP2;1-mCherry construct) (Nelson et al., 2007). This plasma membrane marker was co-expressed with either GFP-VvSWEET7 or GFP-VvSWEET15 constructs, allowing the observation of their co-localization at the plasma membrane revealed by the yellow fluorescence signal.

### Heterologous Expression of *VvSWEET7* and *VvSWEET15* in *Saccharomyces cerevisiae*

The *S. cerevisiae* mutant strain EBY.VW4000 (Wieczorke et al., 1999) was used in this study to functionally characterize VvSWEET7 and VvSWEET15. This strain does not have the capacity to transport monosaccharides and sucrose due to multiple mutations in sugar-sensing and sugar transporter genes. The yeast was grown on rich medium supplemented with maltose (1% yeast extract, 2% peptone, 2% maltose). After transformation by the lithium acetate method (Gietz and Woods, 2002), with the constructions *pYES-DEST52-VvSWEET7* or *pYES-DEST52-VvSWEET15*, the yeast was grown in basic selective medium [0.17% (w/v) yeast nitrogen base, 0.5% (w/v) ammonium sulfate, 2% (w/v) carbon source] supplemented with maltose 2% (w/v) and without uracil for URA3-based selection. For control, the yeast cells were transformed with the empty vector.

### Transport Studies in *S. cerevisiae* With Radiolabeled Sugars

EBY.VW4000 yeast cells, transformed with *pYES-DEST52-VvSWEET7* or *pYES-DEST52-VvSWEET15* (empty pYES-DEST52 for control) were grown in basic selective medium supplemented with 2% maltose at 30°C on a rotatory shaker at 220 rpm up to the exponential-stationary phase. To induce the expression of the target genes, the culture was washed twice in ice-cold sterile water and cultivated in fresh basic selective medium supplemented with 2% galactose at least during 4 h. Then, the cells were harvested by centrifugation and washed twice with ice-cold sterile distilled water and suspended in sterile water. To functionally characterize VvSWEET7 and VvSWEET15 and estimate the initial uptake rates of radiolabeled sugars, 30 μl of cell suspension were mixed with 15 μl of 100 mM KH_2_PO_4_ buffer at pH 5.0 in 1.5 mL microcentrifuge tubes. After 2 min of pre-incubation at 30°C in a thermoblock, the reaction was initiated by the addition of a volume of up to 15 μl of an aqueous solution of radiolabeled glucose (D-[^14^C] glucose) or fructose (D-[^14^C] fructose) with a specific activity of 150 dpm nmol^-1^. Similarly, to determine sucrose initial transport rates, a volume of up to 15 μl of an aqueous solution of radiolabeled sucrose ([^14^C]-sucrose) with a specific activity of 500 (for final concentrations between 7.5 and 50 mM) or 250 dpm nmol^-1^ (for final concentrations between 75 and 125 mM) was used. Potential competitive inhibitors or CCCP (carbonyl cyanide m-chlorophenylhydrazone) were added to the reaction mixture before the addition of the radiolabeled sugar for transport specificity and energetics assessment, respectively. After 3 min, the reaction was stopped by dilution with 1 mL of ice-cold water. Then, cells were washed twice with ice-cold water and 1 mL of scintillation fluid added for complete cell membrane disruption and radioactivity measurements. The radioactivity was then measured in a scintillation counter (Packard Tri-Carb 2200 CA). D-[^14^C] glucose (287 mCi mmol^-1^), D-[^14^C] fructose (316 mCi mmol^-1^), and [^14^C] sucrose (592 mCi mmol^-1^) were obtained from American Radiolabeled Chemicals (St. Louis, MO, USA). For every radiolabeled sugar transport experiment, three independent experimental repetitions, each one consisting of an independent VvSWEET7-overexpressing and respective control yeast growth and subsequent radiolabeled sugar uptake were performed. Also, each experimental repetition was performed with three technical replicates.

### Measurement of Proton Pumping Activity of the Yeast Plasma Membrane ATPase

EBY.VW4000 yeast cells, transformed with *pYES-DEST52-VvSWEET7* and control ones (harboring the empty vector) were washed with deionized water and suspended in water (20 mg mL^-1^) at room temperature under stirring for 3 h to induce starvation. For each experimented condition, 15 mg (D.W.) of yeast cell suspensions grown until OD_600_ = 0.8 was placed in a water-jacketed chamber with a total volume of 5 mL of non-buffered water. The suspension was mixed with a magnetic stirrer, and the temperature-regulated circulating water was at 30°C. Changes in pH were detected with a combination electrode (PHC-4000-8 RadioMeter) attached to a sensitive pH meter (PHM82 Standard pH Meter) and recorder (KIPP & ZONEN) with scale expander, as described by Serrano (1980). A concentration of 45 mM of different sugars (glucose, fructose, galactose, and sucrose) were used to activate the proton pump. Calibration was performed through the addition of 100 nmol HCl to the cell suspension. For proton pumping activity analysis, four experimental repetitions were performed, each one consisting of an independent VvSWEET7-overexpressing and control yeast growth and a subsequent sugar-induced pH variation analysis.

### Statistical Analysis

To test if the data were normally distributed, the Shapiro-Wilk normality test was used, while the homogeneity of variances was confirmed using Bartlett’s tests using Prism v. 6 (GraphPad Software, Inc.). Subsequently, the results were statistically verified by analysis of variance tests (one-way ANOVA) or Student’s t-test using Prism v. 6 (GraphPad Software, Inc.). Post-hoc multiple comparisons were performed using the HSD Tukey test. Throughout the results, different letters denote statistical differences between columns and are presented in a progressive order from the highest to the lowest value, and asterisks indicate statistical significance.

## Results

### Effect of *B. cinerea* Infection on the Expression Profile of Grapevine *VvSWEET* Genes

Grape berry clusters showed clear symptoms of infection at the green (EL32), veraison (EL35), and fully mature stages (EL38), which were very similar among infected clusters that were confirmed by amplification by qPCR of specific fungal genomic DNA as previously reported in **Figure 2** by Coelho et al. (2019). Also, visual symptoms of infection can be seen in **Supplementary Figure 1**.

**Figure1:**
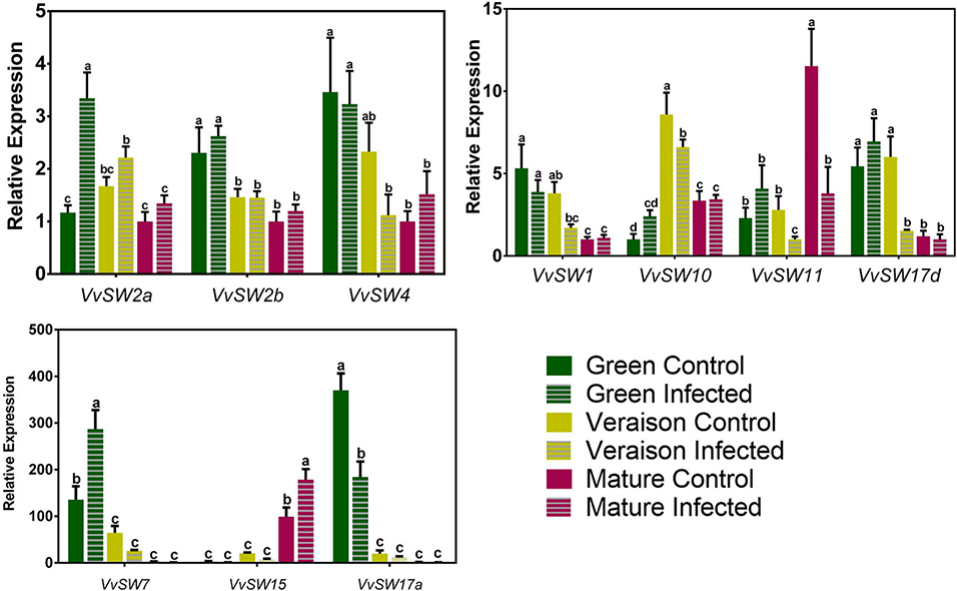
Expression profile of *VvSWEET* genes that are expressed in the grape berry, performed by real-time qPCR in infected (striped bars) and control (solid bars) berries, collected at three different developmental stages (green, veraison and mature). Relative expression for each gene was calculated by the Bio-Rad^®^ CFX Manager 2.0 Software and was determined against the sample with the lowest expression level, which was set to 1. For each of the three biological replicates, after RNA extractions and cDNA synthesis, an independent qPCR analysis was performed with three internal technical replicates. Values are the mean ± SD. (one-way ANOVA with Tukey’s post-test; different letters denote statistical differences between columns).

**Figure 2:**
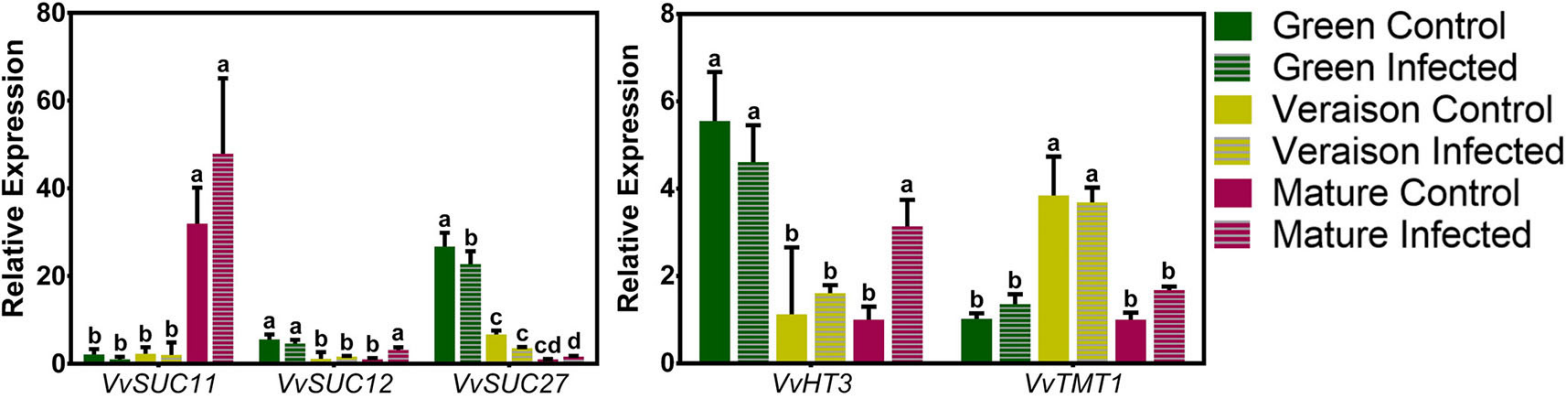
Expression profile of some *VvSUCs* and *VvHTs* genes, highly expressed in the grape berry, performed by real-time PCR in infected (striped bars) and control (solid bars) berries, collected at three different developmental stages (green, veraison, and mature). Relative expression for each gene was calculated by the Bio-Rad^®^ CFX Manager 2.0 Software and was determined against the sample with the lowest expression level, which was set to 1. For each of the three biological replicates, after RNA extractions and cDNA synthesis, an independent qPCR analysis was performed with three internal technical replicates. Values are the mean ± SD. (one-way ANOVA with Tukey’s post-test; different letters denote statistical differences between columns).

**Figure 1** shows that distinctive expression patterns along grape berry development were observed for each *VvSWEET* gene. While the transcript levels of *VvSWEET11* and *VvSWEET15* increased along development, the expression of *VvSWEET1*, *VvSWEET2b*, *VvSWEET4*, *VvSWEET7*, *VvSWEET17a*, and *VvSWEET17d* decreased from green to mature stage. The transcript levels of *VvSWEET10* peaked at veraison, and *VvSWEET2a* gene expression was similar during grape berry development. In each stage, the gene expression in whole berries was compared between control non-infected grapes (solid bars in **Figure 1**) and *B. cinerea*–infected berries (striped bars in **Figure 1**). As can be seen, *B. cinerea* infection up-regulated *VvSWEET2a* and *VvSWEET7* expression at the green stage and *VvSWEET15* expression at the mature stage, while down-regulated *VvSWEET17a* expression at the green stage, *VvSWEET10* and *VvSWEET17d* expression at the veraison stage, and *VvSWEET11* expression at the mature stage. Interestingly, down-regulation of *VvSWEET* genes occurred specifically in the developmental stages where the gene was most expressed in normal conditions. Expression of *VvSWEET1*, *VvSWEET2b*, and *VvSWEET4* was not modified by the infection. The expression of most *VvSWEET* genes, including *VvSWEET7*, in *Botrytis*-elicited grape berry cell suspensions originating from the pulp of berries from Cabernet Sauvignon berries, was modified in a similar way to the changes observed in infected grape berries from the field experiment when they occurred, with the exception of only *VvSWEET1* and *VvSWEET17a*, whose expression was repressed or unaltered, respectively (**Supplementary Figure 2**). These results confirm a similar *B. cinerea* effect also at more controlled conditions. The effect of *B. cinerea* infection on the expression profile of *VvSUC11* (grapevine sucrose transporter 11), *VvSUC12* (grapevine sucrose transporter 12), *VvSUC27* (grapevine sucrose transporter 27), *VvHT3* (grapevine hexose transporter 3), and *VvTMT1* (grapevine tonoplast monosaccharide transporter 1)—prominent members of the Major Facilitator Superfamily (MFS), is shown in **Figure 2**. As previously shown (Afoufa-Bastien et al., 2010), *VvSUC11* is mostly expressed in mature berries, while the transcript levels of *VvSUC12*, *VvSUC27*, and *VvHT3* are more abundant at the green stage. *VvTMT1* expression peaked at veraison. From all studied genes, only *VvHT3* was responsive to *B. cinerea* infection, which caused a 3-fold up-regulation at the mature stage.

### *In* *Silico* Characterization of *VvSWEET7* and *VvSWEET15*

The sequences of both *VvSWEET7* (GSVIVG01019601001) and *VvSWEET15* (GSVIVG01000938001) present two PFAM motif PF03083/MtN3_slv and are predicted to have seven transmembrane domains targeted to the plasma membrane. PLACE (Higo et al., 1999) and PlantPAN 3.0 databases (Chow et al., 2019) (**Supplementary Tables 2** and **3**) revealed that *VvSWEET7* promoter sequence (2kbp upstream) has several biotic stress-related cis-acting elements, such as WRKY71OS and GT1GMSCAM4; and also some sugar responsive elements as WBOXHVIS01, MYBGAHV, or SUR2STPAT21, a sucrose responsive element, a motif conserved among genes regulated by sucrose (**Supplementary Tables 2** and **3**). Also, several abiotic and hormone responsive cis-acting elements were localized. Several responsive elements that were identified in the *VvSWEET7* promoter region were also localized in the promoter region of the *VvSWEET15* gene, as the biotic stress related WRKY71OS and GT1GMSCAM4, or the abiotic stress related cis-acting element MYCCONSENSUSAT. However, only a few sugar responsive elements were identified in the promoter region of *VvSWEET15* gene, as the SBOXATRBCS element. When compared with other *VvSWEETs*, *VvSWEET15* presents fewer sugar responsive elements in its promoter region (**Supplementary Table 4**).

### Sub-Cellular Localization of VvSWEET7 and VvSWEET15

**Figure 3:**
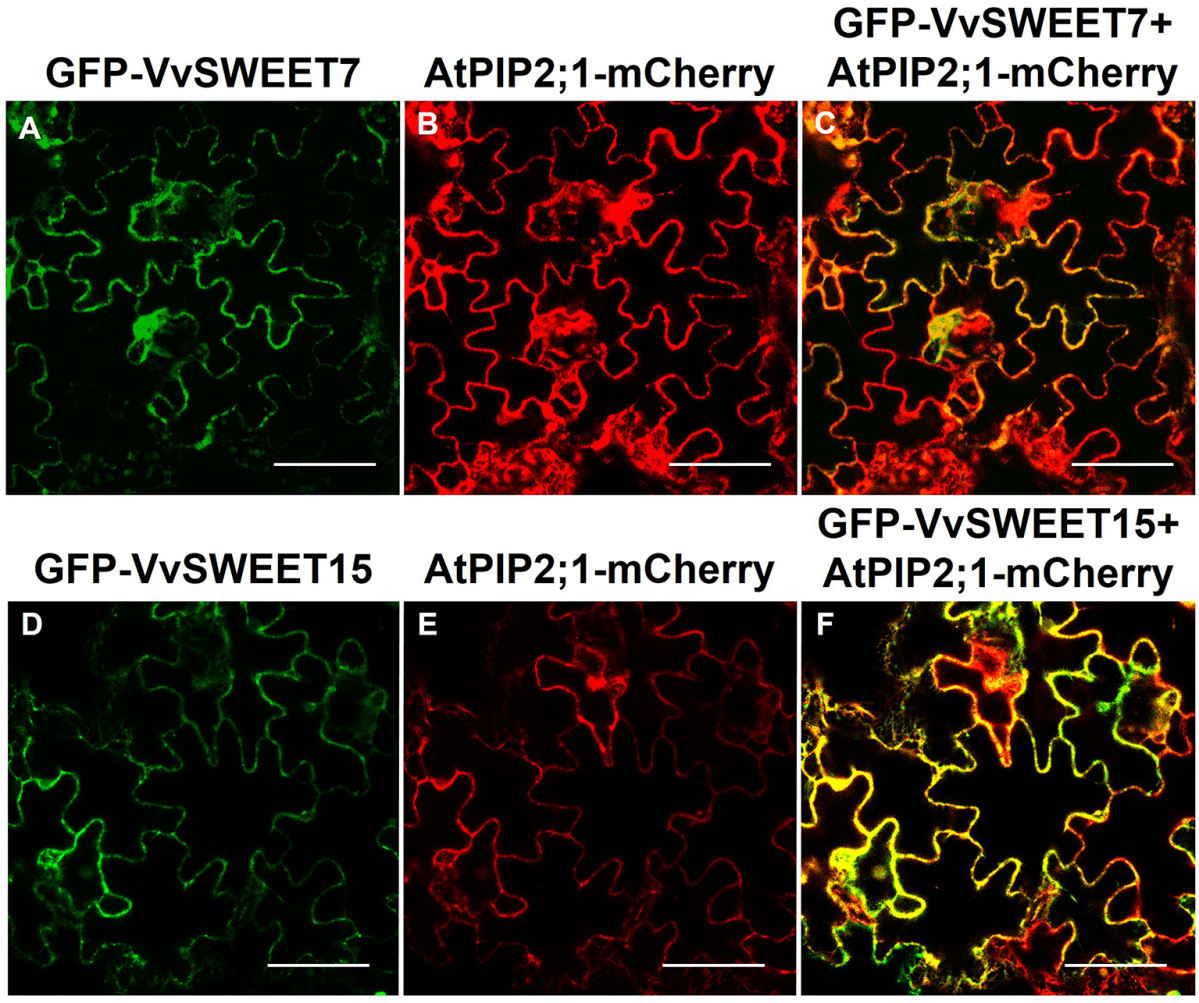
Sub-cellular localization of VvSWEET7 (sub-figures **A–C**) and VvSWEET15 (sub-figures **D–F**) in tobacco leaves. Plasma membrane aquaporin AtPIP2;1 was used as a plasma membrane marker (positive control) (Nelson et al., 2007). Both GFP-VvSWEET7 and GFP-VvSWEET15 localize to the plasma membrane of leaf epidermis cells, as demonstrated by the yellow fluorescence signal observed by confocal microscopy resulting from co-localization of either GFP-VvSWEET7 or GFT-VvSWEET15 fusion proteins with the plasma membrane marker AtPIP2;1-mCherry. All pictures are representative of 3 different replicates. Bar = 100 µm.

VvSWEET7-GFP and VvSWEET15-GFP fusion proteins were transiently expressed in *Nicotiana benthamiana* epidermal cells and co-localization studies with the fusion protein AtPIP2.1-RFP, an aquaporin targeted to the plasma membrane, revealed that both VvSWEET7 and VvSWEET15 localize to the plasma membrane (**Figure 3**).

### Functional Characterization of VvSWEET7 by Heterologous Expression in *Saccharomyces cerevisiae*

**Figure 4:**
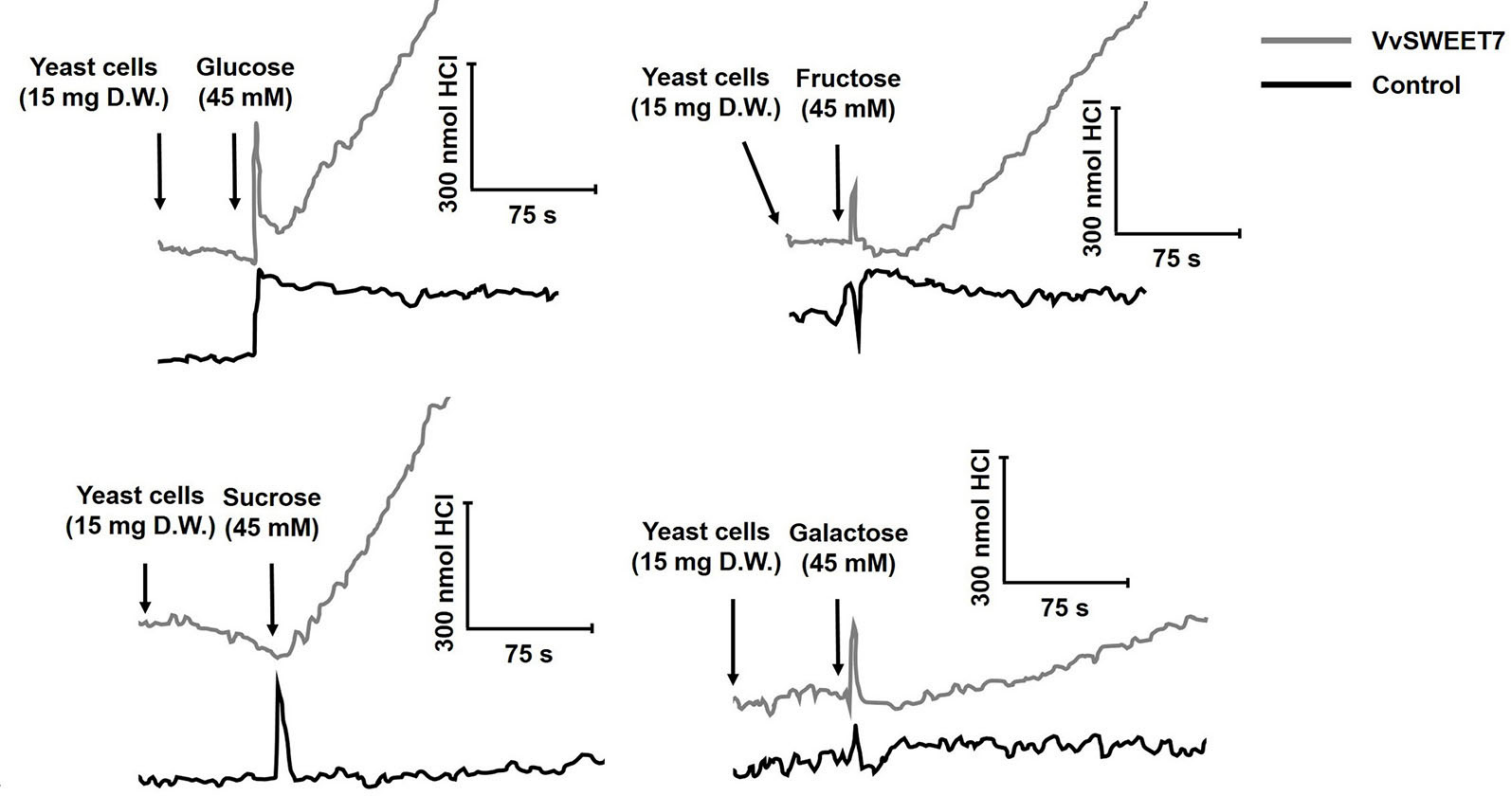
Representative experiments of the activation of the plasma membrane H^+^-ATPase in suspensions of VvSWEET7-expressing EBY.VW4000 yeast cells and controls (empty vector) induced by mono- and disaccharides. For proton pumping activity analysis, four experimental repetitions were performed, each one consisting of an independent VvSWEET7-overexpressing and control yeast growth and subsequent sugar-induced pH variation analysis. All illustrations are representative of 4 different replicates.

The *hxt*-null yeast strain EBY-VW4000 was transformed with pYES-DEST52 containing the cloned *VvSWEET7* cDNA under the control of the galactose-inducible *GAL1* promoter. The first evidence for the involvement of a sugar transport system was provided from the studies of the P-type ATPase activity after the addition of different sugars to suspensions of *VvSWEET7*-transformed cells (**Figure 4**). As can be seen, a clear acidification signal was recorded after addition of glucose, fructose, or sucrose to yeast cells harboring the construct *pYES-DEST52-VvSWEET7* that was not observed in suspensions of yeast cells transformed with the empty vector. The acidification signal after the addition of galactose was less evident. These results suggested that VvSWEET7 is capable of transporting both mono- and disaccharides that, once inside the cells, are catabolized into ATP that activates the proton pump.

**Figure 5:**
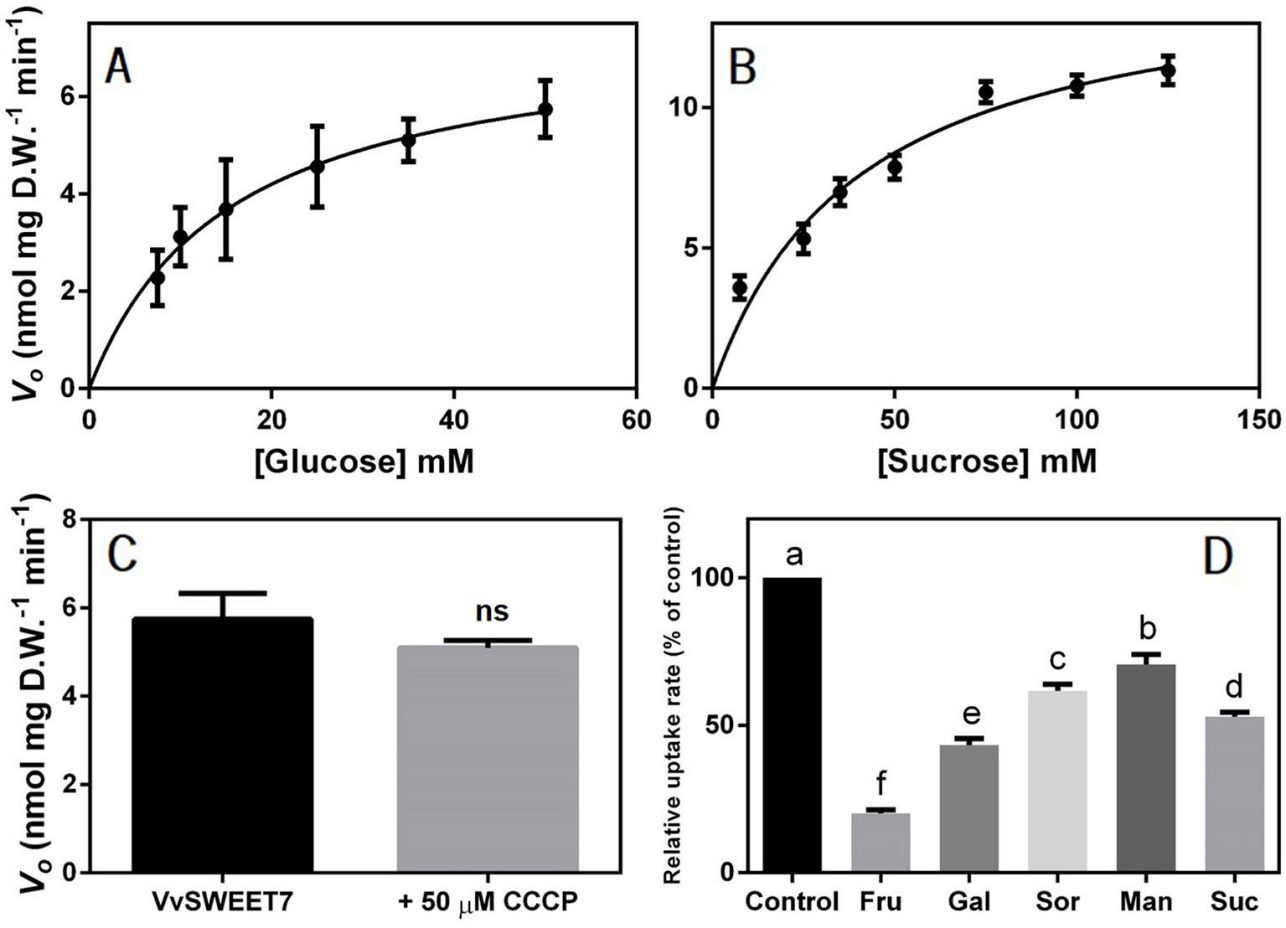
Concentration dependence of the initial uptake rates of D-[^14^C] glucose **(A)** and [^14^C] sucrose **(B)** in VvSWEET7-expressing EBY.VW4000 cells at pH 5.0. **(C)** Effect of 50 µM of the protonophore CCCP (carbonyl cyanide m-chlorophenylhydrazone) on VvSWEET7-mediated uptake of 25 mM D-[^14^C] glucose. **(D)** Competitive inhibition of VvSWEET7-mediated glucose uptake: substrate specificity of VvSWEET7 – fructose, galactose, sorbitol, mannitol, and sucrose at a concentration of 500 mM was added immediately before the addition of 25 mM of radiolabeled D-[^14^C] glucose. The duration of all uptake experiments was 3 min. All values are the mean ± SD of three independent experimental repetitions, each one consisting of an independent VvSWEET7-overexpressing and control yeast growth and subsequent radiolabeled sugar uptake with three technical replicates. (one-way ANOVA with Tukey’s post-test; different letters denote statistical differences between columns). ns, non significant.

The uptake of radiolabeled substrates was also performed and, as shown in **Figure 5**, both the initial uptake rates of 7.5-50 mM D-[^14^C]-glucose and 7.5-125 mM [^14^C]-sucrose followed Michaelis-Menten kinetics, suggesting carrier-mediated transport for both substrates. The kinetic parameters were as follows: K_m_, 15.42 mM glucose and V_max_, 7.4 nmol glucose mg D.W.^-1^ min^-1^ and K_m_, 40.08 mM sucrose and V_max_ 15.12 nmol sucrose mg D.W.^-1^ min^-1^ (**Figures 5A**). Moreover, the addition of 50 μM of the protonophore carbonyl cyanide m-chlorophenyl hydrazine (CCCP) did not inhibit the uptake of 25 mM D-[^14^C]-glucose at pH 5.0, suggesting the transport mechanism was not dependent on the proton gradient (**Figure 5**).

To assess the substrate specificity of VvSWEET7, the uptake rate of 25 mM of D-[^14^C]-glucose was determined in the presence of putative competitive inhibitors of glucose transport, such as other monosaccharides, disaccharides, and polyols at a concentration 20-fold higher than that of D-[^14^C]-glucose (**Figure 5**). Fructose inhibited radiolabeled glucose uptake by 79%, galactose by 56%, sucrose by 47%, mannitol by 29%, and sorbitol by 38%. These results suggest that VvSWEET7 has a broad transport capacity, including for sugar-alcohols.

Attempts were also made to assess the ability of EBY-VW4000 cells expressing VvSWEET15 to transport sugars (glucose, fructose, and sucrose), but no VvSWEET15-mediated sugar transport was detected in any case (data not shown), suggesting that VvSWEET15 may not have such a function.

## Discussion

The proteins encoded by *VvSWEET7* and *VvSWEET15* are likely to play important roles during fruit development and ripening. Our results, obtained in the variety Trincadeira, typically susceptible to *Botrytis* infection, are consistent with previous RNAseq results in Corvina cv. berries (Zenoni et al., 2010) regarding the expression profile throughout berry development, showing that *VvSWEET7* expression in berries peaks at the green stage and *VvSWEET15* at the mature stage. In the present study, we showed that the transcription of these 2 genes in response to *Botrytis* infection was up-regulated in those stages when the basal gene expression is high. The transcript levels of *VvSWEET2a* were also substantially increased at the green stage in response to *Botrytis* infection. In agreement with this observation, the expression of *VvSWEET2a* and *VvSWEET7* in grape leaves increased 72 h after foliar inoculation with *Botrytis* (Chong et al., 2014). Still, no such response had been previously demonstrated in grape berry infection with *Botrytis*, and different plant tissues could, in theory, have different *SWEET* transcriptional responses to *Botrytis*.

Transcriptional reprograming of the expression of *SWEET* genes in response to *Botrytis* infection has also been reported in other plant species. The *Arabidopsis AtSWEET4*, *AtSWEET15* and *AtSWEET17* (Chen et al., 2010) and tomato *SlSWEET15* (Asai et al., 2016) are up-regulated by the infection. Other fungal pathogens such as *Golovinomyces cichoracearum* and mycorrhizal fungus as *Rhizophagus irregularis* (Ferrari et al., 2007; Chen et al., 2010; Manck-Götzenberger and Requena, 2016), as well as bacterial pathogens (Chen et al., 2010), are also known to modulate host *SWEET* gene expression. Transcriptional activator-like (TAL) effectors of *Xanthomonas oryzae* pv. *oryzae* induce rice *OsSWEET11*, *OsSWEET13,* and *OsSWEET14* expression (Chen et al., 2010; Zhou et al., 2014). Cassava *MeSWEET10a* and citrus *CsSWEET1* are also induced by *Xanthomonas* (Cohn et al., 2014; Hu et al., 2014). Other bacteria including *Pseudomonas syringae* induced several *AtSWEET* genes in infected leaves of *Arabidopsis* (Chen et al., 2010).

Induction of SWEET transporters by pathogens has been linked with higher susceptibility to pathogen-induced disease, as pathogens can explore these transporters to obtain sugars by SWEET-promoted leakage of sugars into the apoplastic space (Chen et al., 2010; Cohn et al., 2014). However, this correlation has not always been observed. In *Arabidopsis* roots, *AtSWEET2* gene expression was induced more than 10-fold during *Pythium* infection but *atsweet2*-knockout mutants were more susceptible to infection (Chen et al., 2015c). Also, gene expression of *IbSWEET10* was significantly up-regulated in sweet potato infected with *Fusarium oxysporum* and overexpression of the gene improved host resistance (Li et al., 2017). It has been proposed that sugar remobilization can trigger signaling cascades that activate defense mechanisms in plants (Gebauer et al., 2017). In this regard, glucose and sucrose-mediated induction of defense-related secondary metabolism has been reported (Xiao et al., 2000; Morkunas et al., 2005; Solfanelli et al., 2006; Dao et al., 2011; Kim and Hwang, 2014; Tonnessen et al., 2014). As the grape berry is more resistant to *Botrytis* attacks during its green stage (Goetz et al., 1999), it is reasonable to doubt that the overexpression of *VvSWEET2a* and *VvSWEET7* is an exploitation by the fungus to facilitate infection, rather suggesting that their overexpression may be a defense-related mechanism.

More information regarding what stimuli, from sugar availability to pathogen attack or hormonal stimuli, could activate the promoters of grape berry SWEET genes can be thoroughly seen in **Supplementary Tables 2**–**4**, where the identification of cis-acting elements was performed initially using PLACE database (Higo et al., 1999) and subsequently confirmed following analysis using PlantPAN3 (Chow et al., 2019).

It has been hypothesized that if *SWEETs* transporters are exploited by the fungus to promote sugar leakage to the apoplastic space, plants can, as a response, induce secondary-active sugar transporters to retrieve that sugar (Fotopoulos et al., 2003; Lemonnier et al., 2014; Yamada et al., 2016). In *Arabidopsis*, the induction of hexose/H^+^ symporters, such as STP1, 4, and 13 may counteract SWEET-mediated secretion induced by bacterial infection (Fotopoulos et al., 2003; Yamada et al., 2016). The sugar transporter AtSTP13 is phosphorylated after the interaction with the flagellin receptor AtFLS2 and its co-receptor receptor kinase 1 AtBAK1, which enhances AtSTP13 monosaccharide uptake activity. Hence, this transporter can compete with bacteria for extracellular sugars (Yamada et al., 2016). In the present study, out of the five secondary active transporters genes highly expressed in the grape berry (Lecourieux et al., 2014), only *VvHT3* was up-regulated at the mature stage in response to infection (**Figure 2**). This putative hexose transporter gene is the most highly expressed member of the *VvHT* family in the mature berry (Afoufa-Bastien et al., 2010), so it is tempting to speculate that it could indeed be recruited to retrieve sugar accumulated in the apoplast in response to infection, but this hypothesis needs further experimental clarification.

Results showed that some *SWEET* members were down-regulated in response to *Botrytis* infection. This was the case of *VvSWEET10*, *VvSWEET11*, *VvSWEET17a*, and *VvSWEET17d* whose transcript levels clearly decreased in the developmental stages where their expression was higher: *VvSWEET17a* at the green stage, *VvSWEET10* and *VvSWEET17d* at veraison, and *VvSWEET11* at the mature stage. It would be interesting to observe if this phenomenon also occurs in berries from grapevine varieties more resistant to *Botrytis*, but such results are so far lacking in the literature. Only a recent study reported a decrease in the expression of *SWEETs* genes upon infection, but in tomato. In *Botrytis*-infected cotyledons, 21 of the 31 tomato *SWEET* genes were down-regulated, including the tomato *VvSWEET10*, *VvSWEET11*, *VvSWEET17a*, and *VvSWEET17d* homologues (Asai et al., 2016). *Botrytis* is capable to silence *Arabidopsis* and tomato genes involved in immunity by producing and translocating small RNAs (sRNAs) that hijack the host RNA interference (RNAi) machinery (Weiberg et al., 2013). However, the significance of the down-regulation of *SWEET* genes during pathogen attack is still puzzling.

Clear-cut co-localization experiments revealed that VvSWEET7 and VvSWEET15 are plasma membrane-bound proteins and were heterologously expressed in an *S. cerevisiae* mutant to study their function. The yeast expressing VvSWEET7 showed the capacity to transport glucose and, remarkably, sucrose. In the presence of the protonophore, CCCP transport capacity of VvSWEET7 was not inhibited, demonstrating facilitated transport, in line with previous reports (review by Chen et al., 2015b). Fructose, mannitol, and sorbitol also inhibited glucose transport, suggesting that besides mono and disaccharides, VvSWEET7 may possibly mediate the transport of polyols. The affinity of SWEET transports has been reported in the mM range from ~9 (AtSWEET1) to ~70mM (AtSWEET12) (Chen et al., 2010; Chen et al., 2012), in line with the results of the present study. So far, in grapevine, only two SWEETs (VvSWEET4 and VvSWEET10) have been functionally characterized as sugar transporters following complementation studies in yeast; contrarily to the present work, kinetics analysis, kinetic parameter determinations, and substrate specificity studies were not performed (Chong et al., 2014; Zhang et al., 2019).

As previously mentioned, the SWEET family is divided into 4 different clades, but within each clade proteins may have different physiological roles (Eom et al., 2015). Considering the few characterized SWEETs, each clade appears to correlate well with the selectivity of each member towards monosaccharides versus disaccharides (Clade I and II prefer hexoses, clade III sucrose) (Chen et al., 2015b). However, in the present study, VvSWEET7 exhibited the capacity to transport both mono and di-saccharides, as previously reported for AtSWEET16 (Klemens et al., 2013).

The possible polyol transport capacity evidenced by VvSWEET7 is so far unique in the SWEET family. The polyol transporter AtPLT5, localized in the plasma membrane of *Arabidopsis*, is able to actively transport a broad-spectrum of substrates such as sorbitol, xylitol, erythritol, or glycerol and also different hexoses, such as glucose and pentoses including ribose, tetroses, and a sugar acid (Reinders et al., 2005; Klepek et al., 2005). Similarly, VvPLT1 (VvPMT5) has been characterized as a polyol transporter that is competitively inhibited by monosaccharides (Conde et al., 2015).

The observed broad range of transported substrates and its high expression in the green stage suggest that VvSWEET7 plays an important role in sugar partitioning during fruit development. At the green fruit stage, sucrose is predominantly translocated to the berry mesocarp cells *via* plasmodesmata (Zhang et al., 2006); however, apoplasmic transport through VvSWEET7 may be also involved. The *Arabidopsis* VvSWEET7 homologue functions as a glucose transport and is expressed mainly in the flower and seed (Chen et al., 2010); the cucumber CsSWEET7b transports glucose and, to a minor degree, mannose and galactose (Li et al., 2017). Interestingly, the tomato VvSWEET7 homologue (SlSWEET6) is also strongly regulated during the early phases of tomato fruit development (Shammai et al., 2018).

However, only a few sugar responsive elements were identified in the promoter region of the *VvSWEET15* gene, as the SBOXATRBCS element. When compared with other *VvSWEETs*, *VvSWEET15* presents fewer sugar responsive elements in its promoter region (**Supplementary Table 4**).

In our experimental conditions, we were not able to demonstrate that VvSWEET15 mediates sugar transport in grapevine, despite the fact that its *Arabidopsis* ortholog, AtSWEET15, is well characterized as a sucrose transporter (Chen et al., 2012). In fact, *VvSWEET15* promoter region has fewer sugar-responsive cis-acting elements than other *VvSWEETs*, and together with the absence of sucrose responsive elements, suggests that VvSWEET15 might actually not be a sugar transporter. Contrarily, in *Arabidopsis*, AtSWEET15 appears to be involved in the remobilization of carbohydrates in senescent leaves as its expression increases by 22-fold during senescence (Quirino et al., 1999). Also, it regulates cell viability under high salinity (Seo et al., 2011) and is also involved, along with AtSWEET11 and AtSWEET12, in the sugar efflux required for seed filling (Chen et al., 2015a). In tomato, the not so well characterized SlSWEET15 showed a similar expression pattern to its grapevine homologue along with fruit development (Shammai et al., 2018), being more expressed in the mature stage.

## Conclusion

Sugar metabolism and mobilization are important players that decide the fate of the ongoing battle between plant and pathogen during infection process. However, despite recent advances, the metabolic signatures and their regulatory nodes, which decide the susceptibility or resistance responses, remain poorly understood. In a variety of grapevine susceptible to *Botrytis* infection, grape berry infection with this pathogen promoted a transcriptional reprograming of the expression of *VvSWEET* genes in sink organs. *VvSWEET7* and *VvSWEET15* are likely to play an important role during fruit development and ripening as they are strongly expressed at the green and mature stage, respectively, and were clearly up-regulated in response to infection. VvSWEET7 was heterologously expressed in yeast and revealed a high-capacity, low-affinity glucose transport with a broad affinity to other substrates like disaccharides and polyols. Previous relevant studies have already addressed the role of key SWEET genes highly expressed in roots, stems, leaves, and nectary tissue (Chen et al., 2010; Chen et al., 2012; Chardon et al., 2013; Guo et al., 2014; Lin et al., 2014; Chong et al., 2014; Zhang et al., 2019), in some cases also demonstrating SWEET response to pathogen attack in such plant tissues; VvSWEET4 and VvSWEET10 have already been demonstrated, by yeast complementation, to be hexose transporters in grapevine. Still, the present study is relevant because: i) functional characterization revealed that VvSWEET7 transports both glucose and sucrose and the affinities for each of the substrates were successfully determined, together with substrate specificity assessment; and ii) the transcriptional reprogramming upon *Botrytis* infection was assessed in the grape berries and notably on a variety susceptible to infection with this pathogen. Considering the advances achieved in this work, together with what was previously known in the literature, the role of SWEETs in plants as friends or foes during pathogenic attack is still a matter of debate.

## Data Availability Statement

All datasets generated/analyzed for this study are included in the article/**Supplementary Material**.

## Author Contributions

RB, AC, and HG conceptualized the work. RB conducted the experiments. DP and AF performed the sample treatment and harvest and the broad promoter analysis. CC and RB conducted the confocal microscope observations. RB, AC, and HG contributed to the analysis of the results. RB, AC, AF, AG, and HG wrote and reviewed the manuscript.

## Funding

The work was supported by National Funds by FCT—Portuguese Foundation for Science and Technology, under the strategic programmes UID/AGR/04033/2019 and UID/BIA/04050/2019. The work was also supported by FCT and European Funds (FEDER/POCI/COMPETE2020) through the research project “MitiVineDrought—Combining “omics” with molecular, biochemical, and physiological analyses as an integrated effort to validate novel and easy-to-implement drought mitigation strategies in grapevine while reducing water use” with ref. PTDC/BIA-FBT/30341/2017 and ref. POCI-01-0145-FEDER-030341, respectively; through the research project “BerryPlastid—Biosynthesis of secondary compounds in the grape berry: unlocking the role of the plastid” with ref. POCI-01-0145-FEDER-028165 and ref. PTDC/BIA-FBT/28165/2017, respectively; and also through the FCT-funded research project “GrapeInfectomics” (PTDC/ASP-HOR/28485/2017). This work was also supported by the project “INTERACT - VitalityWine - ref. NORTE-01-0145-FEDER-000017 – (through FEDER/COMPETE and NORTE2020/CCDR-N). AC was supported with a post-doctoral fellow of the mentioned INTERACT/VitalityWine project with ref. BPD/UTAD/INTERACT/VW/218/2016 and by a post-doctoral researcher contract/position within the project “MitiVineDrought” (PTDC/BIA-FBT/30341/2017 and POCI-01-0145-FEDER-030341). RB was supported with a PhD student grant (PD/BD/113616/2015) under the Doctoral Programme ‘Agricultural Production Chains—from fork to farm’ (PD/00122/2012) funded by FCT. This work also benefited from the networking activities within the European Union-funded COST Action CA17111—”INTEGRAPE—Data Integration to maximize the power of omics for grapevine improvement.”

## Conflict of Interest

The authors declare that the research was conducted in the absence of any commercial or financial relationships that could be construed as a potential conflict of interest.

## References

[B1] Afoufa-Bastien D. Medici A. Jeauffre J. Coutos-Thevenot P. Lemoine R. Atanassova R. (2010). The *Vitis vinifera* sugar transporter gene family: phylogenetic overview and macroarray expression profiling. BMC Plant Biol. 10, 245. 10.1186/1471-2229-10-245 21073695 PMC3095327

[B2] Agudelo-Romero P. Erban A. Rego C. Carbonell-Bejerano P. Nascimento T. Sousa L. (2015). Transcriptome and metabolome reprogramming in *Vitis vinifera* cv. Trincadeira berries upon infection with *Botrytis cinerea* . J. Exp. Bot. 66, 1769–1785. 10.1093/jxb/eru517 25675955 PMC4669548

[B3] Antony G. Zhou J. Huang S. Li T. Liu B. White F. (2010). Rice *xa13* recessive resistance to bacterial blight is defeated by induction of the disease susceptibility gene *Os-11N3* . Plant Cell 22, 3864–3876. 10.1105/tpc.110.078964 21098734 PMC3015117

[B4] Asai Y. Kobayashi Y. Kobayashi I. (2016). Increased expression of the tomato *SISWEET15* gene during grey mold infection and the possible involvement of the sugar efflux to apoplasm in the disease susceptibility. J. Plant Pathol. Microbiol. 7, 329. 10.4172/2157-7471.1000329

[B5] Azevedo H. Conde C. Gerós H. Tavares R. M. (2006). The non-host pathogen *Botrytis cinerea* enhances glucose transport in *Pinus pinaster* suspension-cultured cells. Plant Cell Physiol. 47, 290–298. 10.1093/pcp/pci248 16407393

[B6] Baker R. F. Leach K. A. Braun D. M. (2012). SWEET as sugar: new sucrose effluxers in plants. Mol. Plant 5, 766–768. 10.1093/mp/SSS054 22815540

[B7] Berger S. Sinha A. K. Roitsch T. (2007). Plant physiology meets phytopathology: plant primary metabolism and plant–pathogen interactions. J. Exp. Bot. 58, 4019–4026. 10.1093/jxb/erm298 18182420

[B8] Bezrutczyk M. Hartwig T. Horschman M. Char S. N. Yang J. Yang B. (2018). Impaired phloem loading in *zmsweet13a,b,c* sucrose transporter triple knock-out mutants in *Zea mays* . New Phytol. 218, 594–603. 10.1111/nph.15021 29451311

[B9] Blanco-Ulate B. Vincenti E. Powell A. L. Cantu D. (2013). Tomato transcriptome and mutant analyses suggest a role for plant stress hormones in the interaction between fruit and *Botrytis cinerea* . Front. Plant Sci. 4, 142. 10.3389/fpls.2013.00142 23717322 PMC3653111

[B10] Blanco-Ulate B. Amrine H. Collins S. Rivero M. Vicente R. Morales-Cruz A. (2015). Developmental and metabolic plasticity of white-skinned grape berries in response to *Botrytis cinerea* during noble rot. Plant Physiol. 169 (4), 2422–2443. 10.1104/pp.15.00852 26450706 PMC4677888

[B11] Calderón A. A. Zapata J. M. Barceló A. R. (1994). Differential expression of a cell wall-localized peroxidase isoenzyme capable of oxidizing 4-hydroxystilbenes during the cell culture of grapevine (*Vitis vinifera* cv. Airen and Monastrell). Plant Cell Tissue Organ Cult. 37, 121–127. 10.1007/BF00043605

[B12] Cantu D. Vicente A. R. Greve L. C. Dewey F. M. Bennett A. B. Labavitch J. M. (2008). The intersection between cell wall disassembly, ripening, and fruit susceptibility to *Botrytis cinerea* . Proc. Natl. Acad. Sci. U.S.A. 105, 859–864. 10.1073/pnas.0709813105 18199833 PMC2242701

[B13] Cantu D. Blanco-Ulate B. Yang L. Labavitch J. M. Bennett A. B. Powell A. L. T. (2009). Ripening-regulated susceptibility of tomato fruit to *Botrytis cinerea* requires *NOR* but not *RIN* or ethylene. Plant Physiol. 150, 1434–1449. 10.1104/pp.109.138701 19465579 PMC2705034

[B14] Centeno D. C. Osorio S. Nunes-Nesi A. Bertolo A. L. Carneiro R. T. Araújo W. L. (2011). Malate plays a crucial role in starch metabolism, ripening, and soluble solid content of tomato fruit and affects postharvest softening. Plant Cell 23, 162–184. 10.1105/tpc.109.072231 21239646 PMC3051241

[B15] Chardon F. Bedu M. Calenge F. Klemens P. A. Spinner L. Clement G. (2013). Leaf fructose content is controlled by the vacuolar transporter *SWEET17* in *Arabidopsis* . Curr. Biol. 23, 697–702. 10.1016/j.cub.2013.03.021 23583552

[B16] Chen L. Q. Hou B. H. Lalonde S. Takanaga H. Hartung M. L. Qu X. Q. (2010). Sugar transporters for intercellular exchange and nutrition of pathogens. Nature 468, 527–532. 10.1038/nature09606 21107422 PMC3000469

[B17] Chen L. Q. Qu X. Q. Hou B. H. Sosso D. Osorio S. Fernie A. R. (2012). Sucrose efflux mediated by SWEET proteins as a key step for phloem transport. Science 335, 207–211. 10.1126/science.1213351 22157085

[B18] Chen L. Q. Lin I. W. Qu X. Q. Sosso D. McFarlane H. E. Londono A. (2015a). A cascade of sequentially expressed sucrose transporters in the seed coat and endosperm provides nutrition for the *Arabidopsis* embryo. Plant Cell 27, 607–619. 10.1105/tpc.114.134585 25794936 PMC4558658

[B19] Chen L. Q. Cheung L. S. Feng L. Tanner W. Frommer W. B. (2015b). Transport of sugars. Annu. Rev. Biochem. 84, 865–894. 10.1146/annurev-biochem-060614-033904 25747398

[B20] Chen H. Y. Huh J. H. Yu Y. C. Ho L. H. Chen L. Q. Tholl D. (2015c). The *Arabidopsis* vacuolar sugar transporter SWEET2 limits carbon sequestration from roots and restricts *Pythium* infection. Plant J. 83, 1046–1058. 10.1111/tpj.12948 26234706

[B21] Chong J. Piron M. C. Meyer S. Merdinoglu D. Bertsch C. Mestre P. (2014). The SWEET family of sugar transporters in grapevine: VvSWEET4 is involved in the interaction with *Botrytis cinerea* . J. Exp. Bot. 65, 6589–6601. 10.1093/jxb/eru375 25246444

[B22] Chow C. Lee T. Hung Y. Li G. Tseng K. Liu Y. (2019). PlantPAN3.0: a new and updated resource for reconstructing transcriptional regulatory networks from ChIP-seq experiments in plants. Nucleic Acids Res. 8, 1155–1163. 10.1093/nar/gky1081Chu PMC6323957 30395277

[B23] Chu Z. Fu B. Yang H. Xu C. Li Z. Sanchez A. (2006). Targeting *xa13,* a recessive gene for bacterial blight resistance in rice. Theor. Appl. Genet. 112, 455–461. 10.1007/s00122-005-0145-6 16328230

[B24] Clark J. I. M. Hall J. L. (1998). Solute transport into healthy and powdery mildew-infected leaves of pea and uptake by powdery mildew mycelium. New Phytol. 140, 261–269. 10.1046/j.1469-8137.1998.00263.x 33862850

[B25] Coelho J. Almeida-Trapp M. Pimentel D. Soares F. Reis P. Rego C. (2019). The study of hormonal metabolism of Trincadeira and Syrah cultivars indicates new roles of salicylic acid, jasmonates, ABA and IAA during grape ripening and upon infection with *Botrytis cinerea* . Plant Sci. 283, 266–277. 10.1016/j.plantsci.2019.01.024 31128697

[B26] Cohn M. Bart R. S. Shybut M. Dahlbeck D. Gomez M. Morbitzer R. (2014). *Xanthomonas axonopodis* virulence is promoted by a transcription activator-like effector-mediated induction of a SWEET sugar transporter in cassava. Mol. Plant Microbe Interact. 27, 1186–1198. 10.1094/MPMI-06-14-0161-R 25083909

[B27] Conde A. Regalado A. Rodrigues D. Costa M. J. Blumwald E. Chaves M. M. (2015). Polyols in grape berry: transport and metabolic adjustments as a physiological strategy for water-deficit stress tolerance in grapevine. J. Exp. Bot. 66 (3), 889–906. 10.1093/jxb/eru446 25433029

[B28] Coombe B. (1995). Growth stages of the grapevine: adoption of a system for identifying grapevine growth stages. Aust. J. Grape Wine R. 1, 104–110. 10.1111/j.1755-0238.1995.tb00086.x

[B29] Dalmais B. Schumacher J. Moraga J. Le Pêcheur P. Tudzynski B. Collado I. G. (2011). The *Botrytis cinerea* phytotoxin botcinic acid requires two polyketide synthases for production and has a redundant role in virulence with botrydial. Mol. Plant Pathol. 12, 564–579. 10.1111/j.1364-3703.2010.00692.x 21722295 PMC6640383

[B30] Dao T. T. Linthorst H. J. Verpoorte R. (2011). Chalcone synthase and its functions in plant resistance. Phytochem. Rev. 10, 397–412. 10.1007/s11101-011-9211-7 21909286 PMC3148432

[B31] Decendit A. Ramawat K. G. Waffo P. Deffieux G. Badoc A. Mérillon J. M. (1996). Anthocyanins, catechins, condensed tannins and piceid production in Vitis vinifera cell bioreactor cultures. Biotechnol. Lett. 18, 659–662. 10.1007/BF00130761

[B32] Dulermo T. Rascle C. Chinnici G. Gout E. Bligny R. Cotton P. (2009). Dynamic carbon transfer during pathogenesis of sunflower by the necrotrophic fungus *Botrytis cinerea*: from plant hexoses to mannitol. New Phytol. 183, 1149–1162. 10.1111/j.1469-8137.2009.02890.x 19500266

[B33] Elad Y. Williamson B. Tudzynski P. Delen N. (2004). “ *Botrytis* spp. and diseases they cause in agricultural systems-an introduction,” in Botrytis: biology, pathology and control. Eds. Elad Y. Williamson B. Tudzynski P. Delen N. (Dordrecht, the Netherlands: Kluwer), 1–27.

[B34] Elmer P. A. Michailides T. M. (2004). “Epidemiology of *Botrytis cinerea* in orchard and vine crops,” in Botrytis: biology, pathology and control. Eds. Elad Y. Williamson B. Tudzynski P. Delen N. (Dordrecht, the Netherlands: Kluwer), 1–27.

[B35] Eom J. B. Chen L. Q. Sosso D. Julius B. Lin I. W. Qu X. (2015). SWEETs, transporters for intracellular and intercellular sugar translocation. Curr. Opin. Plant Biol. 25, 53–62. 10.1016/j.pbi.2015.04.005 25988582

[B36] Ferrari S. Galletti R. Denoux C. De Lorenzo G. Ausubel F. M. Dewdney J. (2007). Resistance to *Botrytis cinerea* induced in *Arabidopsis* by elicitors is independent of salicylic acid, ethylene, or jasmonate signaling but requires PHYTOALEXIN DEFICIENT3. Plant Physiol. 144, 367–379. 10.1104/pp.107.095596 17384165 PMC1913806

[B37] Fotopoulos V. Gilbert M. J. Pittman J. K. Marvier A. C. Buchanan A. J. Sauer N. (2003). The monosaccharide transporter gene, AtSTP4, and the cell-wall invertase, Atbetafruct1, are induced in *Arabidopsis* during infection with the fungal biotroph *Erysiphe cichoracearum* . Plant Physiol. 132, 821–829. 10.1104/pp.103.021428 12805612 PMC167022

[B38] Gebauer P. Korn M. Engelsdorf T. Sonnewald U. Koch C. Voll L. M. (2017). Sugar accumulation in leaves of *Arabidopsis sweet11/sweet12* double mutants enhances priming of the salicylic acid-mediated defense response. Front. Plant Sci. 8, 1378. 10.3389/fpls.2017.01378 28848581 PMC5550771

[B39] Gietz R. D. Woods R. A. (2006). “Yeast transformation by the LiAc/SS carrier DNA/PEG,” in Yeast protocols. Ed. Xiao W. (Totowa, NJ: Humana Press), 107–120. 10.1385/1-59259-958-3:107 16118429

[B40] Glazebrook J. (2005). Contrasting mechanisms of defense against biotrophic and necrotrophic pathogens. Annu. Rev. Phytopathol. 43, 205–227. 10.1146/annurev.phyto.43.040204.135923 16078883

[B41] Goetz G. Fkyerat A. Metais N. Kunz M. Tabacchi R. Pezet R. (1999). Resistance factors to grey mould in grape berries: identification of some phenolics inhibitors of *Botrytis cinerea* stilbene oxidase. Phytochemistry 52, 759–767. 10.1016/S0031-9422(99)00351-9

[B42] Goossens A. de la Fuente N. Forment J. Serrano R. Portillo F. (2000). Regulation of yeast H^+^-ATPase by protein kinases belonging to a family dedicated to activation of plasma membrane transporters. Mol. Cell. Biol. 20 (20), 7654–7661. 10.1128/mcb.20.20.7654-7661.2000 11003661 PMC86331

[B43] Govrin E. M. Rachmilevitch S. Tiwari B. S. Solomon M. Levine A. (2006). An elicitor from *Botrytis cinerea* induces the hypersensitive response in *Arabidopsis thaliana* and other plants and promotes the gray mold disease. Phytopathology 96, 299–307. 10.1094/PHYTO-96-0299 18944445

[B44] Guo W. J. Nagy R. Chen H. Y. Pfrunder S. Yu Y. C. Santelia D. (2014). SWEET17, a facilitative transporter, mediates fructose transport across the tonoplast of *Arabidopsis* roots and leaves. Plant Physiol. 164, 777–789. 10.1104/pp.113.232751 24381066 PMC3912105

[B45] Haile Z. M. Pilati S. Sonego P. Malacarne G. Vrhovsek U. Engelen K. (2017). Molecular analysis of the early interaction between the grapevine flower and *Botrytis cinerea* reveals that prompt activation of specific host pathways leads to fungus quiescence. Plant Cell Enviro. 40, 1409–1428. 10.1111/pce.12937 28239986

[B46] Hayes M. A. Davies C. Dry I. B. (2007). Isolation, functional characterization, and expression analysis of grapevine (*Vitis vinifera* L.) hexose transporters: differential roles in sink and source tissues. J. Exp. Bot. 58 (8), 1985–1997. 10.1093/jxb/erm061 17452752

[B47] Higo K. Ugawa Y. Iwamoto M. Korenaga T. (1999). Plant cis-acting regulatory DNA elements (PLACE) database: 1999. Nucleic Acids Res. 27, 297–300. 10.1093/nar/27.1.297 9847208 PMC148163

[B48] Hu Y. Zhang J. Jia H. Sosso D. Li T. Frommer W. B. (2014). *Lateral organ boundaries 1* is a disease susceptibility gene for citrus bacterial canker disease. Proc. Natl. Acad. Sci. U.S.A. 111, 521–529. 10.1073/pnas.1313271111 24474801 PMC3910620

[B49] Julius B. T. Leach K. A. Tran T. M. Mertz R. A. Braun D. M. (2017). Sugar transporters in plants: New insights and discoveries. Plant Cell Physiol. 58, 1442–1460. 10.1093/pcp/pcx090 28922744

[B50] Kanno Y. Oikawa T. Chiba Y. Ishimaru Y. Shimizu T. Sano N. (2016). AtSWEET13 and AtSWEET14 regulate gibberellin-mediated physiological processes. Nat. Commun. 7, 13245. 10.1038/ncomms13245 27782132 PMC5095183

[B51] Keller M. Viret O. Cole M. (2003). *Botrytis cinerea* infection in grape flowers: defense reaction, latency and disease expression. Phytopathology 93, 316–322. 10.1094/PHYTO.2003.93.3.316 18944341

[B52] Kim D. S. Hwang B. K. (2014). An important role of the pepper phenylalanine ammonia-lyase gene (*PAL1*) in salicylic acid-dependent signaling of the defence response to microbial pathogens. J. Exp. Bot. 65, 2295–2306. 10.1093/jxb/eru109 24642849 PMC4036500

[B53] Kleemann J. Rincon-Rivera L. J. Takahara H. Neumann U. Van Themaat E. V. L. Van Der Does H. C. (2012). Sequential delivery of host-induced virulence effectors by appressoria and intracellular hyphae of the phytopathogen *Colletotrichum higginsianum* . PloS Pathog. 8 (4), 1002643. 10.1371/journal.ppat.1002643 PMC3320591 22496661

[B54] Klemens P. A. W. Patzke K. Deitmer J. Spinner L. Le Hir R. Bellini C. (2013). Overexpression of the vacuolar sugar carrier *AtSWEET16* modifies germination, growth, and stress tolerance in *Arabidopsis* . Plant Physiol. 163, 1338–1352. 10.1104/pp.113.224972 24028846 PMC3813654

[B55] Klepek Y. S. Geiger D. Stadler R. Klebl F. Landouar-Arsivaud L. Lemoine R. (2005). *Arabidopsis* polyol transporter5, a new member of the monosaccharide transporter-like superfamily, mediates H^+^-symport of numerous substrates, including myo-inositol, glycerol, and ribose. Plant Cell 17, 204–218. 10.1105/tpc.104.026641 15598803 PMC544499

[B56] Kocal N. Sonnewald U. Sonnewald S. (2008). Cell wall-bound invertase limits sucrose export and is involved in symptom development and inhibition of photosynthesis during compatible interaction between tomato and *Xanthomonas campestris* pv *vesicatoria* . Plant Physiol. 148, 1523–1536. 10.1104/pp.108.127977 18784281 PMC2577280

[B57] Kretschmer M. Kassemeyer H. H. Hahn M. (2007). Age-dependent grey mould susceptibility and tissue-specific defence gene activation of grapevine berry skins after infection by *Botrytis cinerea* . J. Phytopathol. 155, 258–263. 10.1111/j.1439-0434.2007.01216.x

[B58] Lecourieux F. Kappel C. Lecourieux D. Serrano A. Torres E. Arce-Johnson P. (2014). An update on sugar transport and signaling in grapevine. J. Exp. Bot. 65 (3), 821–832. 10.1093/jxb/ert394 24323501

[B59] Lemoine R. La Camera S. Atanassova R. Dédaldéchamp F. Allario T. Pourtau N. (2013). Source-to-sink transport of sugar and regulation by environmental factors. Front. Plant Sci. 4, 272. 10.3389/fpls.2013.00272 23898339 PMC3721551

[B60] Lemonnier P. Gaillard C. Veillet F. Verbeke J. Lemoine R. Coutos-Thévenot P. (2014). Expression of *Arabidopsis* sugar transport protein STP13 differentially affects glucose transport activity and basal resistance to *Botrytis cinerea* . Plant Mol. Biol. 85, 473–484. 10.1007/s11103-014-0198-5 24817131

[B61] Li Y. Wang Y. Zhang H. Zhang Q. Zhai H. Liu Q. (2017). The plasma membrane-localized sucrose transporter IbSWEET10 contributes to the resistance of sweet potato to *Fusarium oxysporum* . Front. Plant Sci. 8, 197. 10.3389/fpls.2017.00197 28261250 PMC5306249

[B62] Lin I. W. Sosso D. Chen L. Q. Gase K. Kim S. G. Kessler D. (2014). Nectar secretion requires sucrose phosphate synthases and the sugar transporter SWEET9. Nature 508, 546–549. 10.1038/nature13082 24670640

[B63] Manck-Götzenberger J. Requena N. (2016). Arbuscular mycorrhiza symbiosis induces a major transcriptional reprogramming of the potato SWEET sugar transporter family. Front. Plant Sci. 7, 487. 10.3389/fpls.2016.00487 27148312 PMC4830831

[B64] McClellan W. D. Hewitt W. B. (1973). Early *Botrytis* rot of grapes: time of infection and latency of *Botrytis cinerea* Pers. in *Vitis vinifera* L. Phytopathology 63, 1151–1157. 10.1094/Phyto-63-1151

[B65] Mengiste T. (2012). Plant immunity to necrotrophs. Ann. Rev. Phytopathol. 50 (1), 267–294. 10.1146/annurev-phyto-081211-172955 22726121

[B66] Miedes E. Lorences E. P. (2007). The implication of xyloglucan endotransglucosylase/hydrolase (XTHs) in tomato fruit infection by *Penicillium expansum* Link. A. J. Agric. Food Chem. 55, 9021–9026. 10.1021/jf0718244 17960871

[B67] Morkunas I. Ratajczak L. (2014). The role of sugar signaling in plant defense responses against fungal pathogens. Acta Physiol. Plant 36, 1607–1619. 10.1007/s11738-014-1559-z

[B68] Morkunas I. Marczak Ł. Stachowiak J. Stobiecki M. (2005). Sucrose induced lupine defense against *Fusarium oxysporum.* Sucrose stimulated accumulation of isoflavonoids as a defense response of lupine to *Fusarium oxysporum* . Plant Physiol. Biochem. 43, 363–373. 10.1016/j.plaphy.2005.02.011 15907688

[B69] Murashige T. Skoog F. (1962). A revised medium for rapid growth and bioassays with tobacco tissue cultures. Physiol. Plant 15, 473–497. 10.1111/j.1399-3054.1962.tb08052.x

[B70] Nair N. G. Guilbaud-Oulton. S. Barchia. I. Emmett R. (1995). Significance of carry over inoculum, flower infection and latency on the incidence of *Botrytis cinerea* in berries of grapevines at harvest *Vitis vinifera* in New South Wales. Aust. J. Exp. Agr. 35, 1177–1180. 10.1071/EA9951177

[B71] Nelson B. K. Cai X. Nebenfuhr A. (2007). A multicolored set of *in vivo* organelle markers for co-localization studies in *Arabidopsis* and other plants. Plant J. 51, 1126–1136. 10.1111/j.1365-313X.2007.03212.x 17666025

[B72] Pastenes C. Villalobos L. Ríos N. Reyes F. Turgeon R. Franck N. (2014). Carbon partitioning to berries in water stressed grapevines: the role of active transport in leaves and fruits. Environ. Exp. Bot. 107, 154–166. 10.1016/j.envexpbot.2014.06.009

[B73] Perfect S. E. Hughes H. B. O'Connell R. J. Green J. R. (1999). *Colletotrichum*: a model genus for studies on pathology and fungal–plant interactions. Fungal Genet. Biol. 27, 186–198. 10.1006/fgbi.1999.1143 10441444

[B74] Pezet R. Viret O. Perret C. Tabacchi R. (2003). Latency of *Botrytis cinerea* Pers.: Fr. and biochemical studies during growth and ripening of two grape berry cultivars, respectively susceptible and resistant to grey mould. J. Phytopathol. 151, 208–214. 10.1046/j.1439-0434.2003.00707.x

[B75] Prusky D. Alkan N. Mengiste T. Fluhr R. (2013). Quiescent and necrotrophic lifestyle choice during postharvest disease development. Annu. Rev. Phytopathol. 51, 155–176. 10.1146/annurev-phyto-082712-102349 23682917

[B76] Quirino B. F. Normanly J. Amasino R. M. (1999). Diverse range of gene activity during *Arabidopsis thaliana* leaf senescence includes pathogen-independent induction of defense-related genes. Plant Mol. Biol. 40, 267–278. 10.1023/A:1006199932265 10412905

[B77] Reid K. E. Olsson N. Schlosser J. Peng F. Lund S. T. (2006). An optimized grapevine RNA isolation procedure and statistical determination of reference genes for real-time RT-PCR during berry development. BMC Plant Biol. 6, 27. 10.1186/1471-2229-6-27 17105665 PMC1654153

[B78] Reinders A. Panshyshyn J. A. Ward J. M. (2005). Analysis of transport activity of *Arabidopsis* sugar alcohol permease homolog AtPLT5. J. Biol. Chem. 280, 1594–1602. 10.1074/jbc.M410831200 15525644

[B79] Roitsch T. Balibrea M. E. Hofmann M. Proels R. Sinha A. K. (2003). Extracellular invertase: key metabolic enzyme and PR protein. J. Exp. Bot. 54, 513–524. 10.1093/jxb/erg050 12508062

[B80] Ruiz E. Ruffner H. P. (2002). Immunodetection of *Botrytis*-specific invertase in infected grapes. J. Phytopathol. 150, 76–85. 10.1046/j.1439-0434.2002.00720.x

[B81] Sade D. Brotman Y. Eybishtz A. Cuadros-Inostroza A. Fernie A. R. Willmitzer L. (2013). Involvement of the hexose transporter gene *LeHT1* and of sugars in resistance of tomato to tomato yellow leaf curl virus. Mol. Plant 6, 1707–1710. 10.1093/mp/sst036 23430051

[B82] Seo P. J. Park J. M. Kang S. K. Kim S. G. Park C. M. (2011). An *Arabidopsis* senescence-associated protein SAG29 regulates cell viability under high salinity. Planta 233, 189–200. 10.1007/s00425-010-1293-8 20963606

[B83] Serrano R. (1980). Effect of ATPase inhibitors on the proton pump of respiratory-deficient yeast. Eur. J. Biochem. 105, 419–424. 10.1111/j.1432-1033.1980.tb04516.x 6247154

[B84] Shammai A. Petreikov M. Yeselson Y. Faigenboim A. Moy-Komemi M. Cohen S. (2018). Natural genetic variation for expression of a *SWEET* transporter among wild species of *Solanum lycopersicum* (tomato) determines the hexose composition of ripening tomato fruit. Plant J. 96, 343–357. 10.1111/tpj.14035 30044900

[B85] Siemens J. Gonzalez M. C. Wolf S. Hofmann C. Greiner S. Du Y. (2011). Extracellular invertase is involved in the regulation of clubroot disease in *Arabidopsis thaliana* . Mol. Plant Pathol. 12, 247–262. 10.1111/j.1364-3703.2010.00667.x 21355997 PMC6640435

[B86] Solfanelli C. Poggi A. Loreti E. Alpi A. Perata P. (2006). Sucrose-specific induction of the anthocyanin biosynthetic pathway in *Arabidopsis* . Plant Physiol. 140, 637–646. 10.1104/pp.105.072579 16384906 PMC1361330

[B87] Sparkes I. A. Runions J. Kearns A. Hawes C. (2006). Rapid, transient expression of fluorescent fusion proteins in tobacco plants and generation of stably transformed plants. Nat. Protoc. 1, 2019–2025. 10.1038/nprot.2006.286 17487191

[B88] Tonnessen B. W. Manosalva P. Lang J. M. Baraoidan M. Bordeos A. Mauleon R. (2014). Rice phenylalanine ammonia-lyase gene *OsPAL4* is associated with broad spectrum disease resistance. Plant Mol. Biol. 87, 273–286. 10.1007/s11103-014-0275-9 25515696

[B89] Truernit E. Schmid J. Epple P. Illig J. Sauer N. (1996). The sink-specific and stress-regulated *Arabidopsis STP4* gene: enhanced expression of a gene encoding a monosaccharide transporter by wounding, elicitors, and pathogen challenge. Plant Cell 8, 2169–2182. 10.1105/tpc.8.12.21698/12/2169 8989877 PMC161343

[B90] Vail M. Marois J. (1991). Grape cluster architecture and the susceptibility of berries to *Botrytis cinerea* . Phytopathology 81, 188–191. 10.1094/Phyto-81-188

[B91] van Kan J. A. (2006). Licensed to kill: the lifestyle of a necrotrophic plant pathogen. Trends Plant Sci. 11, 1360–1385. 10.1016/j.tplants.2006.03.005 16616579

[B92] Veloso J. van Kan J. A. (2018). Many shades of grey in *Botrytis*–host plant interactions. Trends Plant Sci. 23 (7), 613–622. 10.1016/j.tplants.2018.03.016 29724660

[B93] Weiberg A. Wang M. Lin F. Zhao H. Zhang Z. Kaloshian I. (2013). Fungal small RNAs suppress plant immunity by hijacking host RNA interference pathways. Science. 342, 118–123. 10.1126/science.1239705 24092744 PMC4096153

[B94] Wieczorke R. Krampe S. Weierstall T. Freidel K. Hollenberg C. P. Boles E. (1999). Concurrent knock-out of at least 20 transporter genes is required to block uptake of hexoses in *Saccharomyces cerevisiae* . FEBS Lett. 464, 123–128. 10.1016/s0014-5793(99)01698-1 10618490

[B95] Wright D. P. Baldwin B. C. Shephard M. C. Scholes J. D. (1995). Source-sink relationships in wheat leaves infected with powdery mildew (I). Alterations in carbohydrate metabolism. Physiol. Molec. Plant Pathol. 47, 237–253. 10.1006/pmpp.1995.1055

[B96] Xiao W. Sheen J. Jang J. C. (2000). The role of hexokinase in plant sugar signal transduction and growth and development. Plant Mol. Biol. 44, 451–461. 10.1023/A:1026501430422 11197321

[B97] Yamada K. Saijo Y. Nakagami H. Takano Y. (2016). Regulation of sugar transporter activity for antibacterial defense in *Arabidopsis* . Science 354, 1427–1430. 10.1126/science.aah5692 27884939

[B98] Yang B. Sugio A. White F. F. (2006). *Os8N3* is a host disease-susceptibility gene for bacterial blight of rice. Proc. Natl. Acad. Sci. U.S.A. 103, 10503–10508. 10.1073/pnas.0604088103 16798873 PMC1502487

[B99] Yang J. Luo D. Yang B. Frommer W. B. Eom J. (2018). SWEET11 and 15 as key players in seed filling in rice. New Phytol. 218, 604–615. 10.1111/nph.15004 29393510

[B100] Zenoni S. Ferrarini A. Giacomelli E. Xumerle L. Fasoli M. Malerba G. (2010). Characterization of transcriptional complexity during berry development in *Vitis vinifera* using RNA-seq. Plant Physiol. 152, 1787–1795. 10.1104/pp.109.149716 20118272 PMC2850006

[B101] Zhang X. Y. Wang X. L. Wang X. F. Xia G. H. Pan Q. H. Fan R. C. (2006). A shift of phloem unloading from symplasmic to apoplasmic pathway is involved in developmental onset of ripening in grape berry. Plant Physiol. 142, 220–232. 10.1104/pp.106.081430 16861573 PMC1557625

[B102] Zhang Z. Zou L. Ren C. Ren F. Wang Y. Fan P. (2019). *VvSWEET10* mediates sugar accumulation in grapes. Genes 10, 255. 10.3390/genes10040255 PMC6523336 30925768

[B103] Zhou Y. Liu L. Huang W. Yuan M. Zhou F. Yongjun L. (2014). Overexpression of *OsSWEET5* in rice causes growth retardation and precocious senescence. PloS One 9, 94210. 10.1371/journal.pone.0094210 PMC3978035 24709840

