# Supplementary material for: VvSWEET7 Is a Mono- and Disaccharide Transporter Up-Regulated in Response to Botrytis cinerea Infection in Grape Berries
Source: Front Plant Sci. 2020 Jan 27;10:1753. doi: 10.3389/fpls.2019.01753 (PMC6996298; doi:10.3389/fpls.2019.01753)
Supplement: Supplementary file 1 [file DataSheet_1.docx]

Supplementary Material

Supplementary table 1 - Sequences of the primers used in the molecular biology approaches of this study.

| **Primer name** | | **Accession Number** | **Sequence** | **Reference** |
| --- | --- | --- | --- | --- |
| *attb-VvSWEET7* | *FW* | GSVIVT01019601001 | GGGGACAAGTTTGTACAAAAAAGCAGGCTCAGCCATGTCTTCTACAGAA | Designed in this study |
|  | *RV* |  | GGGGACCACTTTGTACAAGAAAGCTGGGTTCATGTTCTACGGACCTCAT | Designed in this study |
| *attb-VvSWEET15* | *FW* | GSVIVT01000938001 | GGGGACAAGTTTGTACAAAAAAGCAGGCTTAGAGATGGCTATGGCCATGG | Designed in this study |
|  | *RV* |  | GGGGACCACTTTGTACAAGAAAGCTGGGTTCAAACTGTACTTCCATTTG | Designed in this study |
| *qVvSWEET1* | *FW* | GSVIVT01010015001 | GGATGCTCATCATGCTCTTCA | Chong et al., 2014 |
|  | *RV* |  | AAGCAGAGAGAAGGCAGTTGAG |  |
| *qVvSWEET2a* | *FW* | GSVIVT01014088001 | CGTTCTCTGTTGTTGCCAGTC | Chong et al., 2014 |
|  | *RV* |  | ACCAAGCAGTTTAGGAGAGCA |  |
| *qVvSWEET2b* | *FW* | GSVIVT01021317001 | AATGTCGGGATTATTGACAGCA | Chong et al., 2014 |
|  | *RV* |  | AGGGATGCAACACTCAAATATCC |  |
| *qVvSWEET4* | *FW* | GSVIVT01032489001 | GGCTCGGACTGTGATTGGTA | Chong et al., 2014 |
|  | *RV* |  | ACATGCAGTTCATCACTGTGG |  |
| *qVvSWEET7* | *FW* | GSVIVT01019601001 | ACCGCAGTTGGCATCCTA | Chong et al., 2014 |
|  | *RV* |  | GCAAGGTAGGGAACTGGTGA |  |
| *qVvSWEET10* | *FW* | GSVIVT01008595001 | CCATTCACCATCCTTTGGTTT | Chong et al., 2014 |
|  | *RV* |  | CCACGTAGGGAACAGACTGAA |  |
| *qVvSWEET11* | *FW* | GSVIVT01010993001 | GGGACGTGCATAGAAGCTACA | Chong et al., 2014 |
|  | *RV* |  | GCAGACCCAACCGACTATCTT |  |
| *qVvSWEET15* | *FW* | GSVIVT01000938001 | GGCCAAGAAACAAACTCTCAAA | Chong et al., 2014 |
|  | *RV* |  | GCCACTGAGAATGAAGCACAG |  |
| *qVvSWEET17a* | *FW* | GSVIVT01035138001 | GGTTTTGGTGTGGTTGTTGAA | Chong et al., 2014 |
|  | *RV* |  | AGCTAGAAACCCCACATCCAA |  |
| *qVvSWEET17d* | *FW* | GSVIVT01031170001 | CTGGCGGCTTACTTGTCCT | Chong et al., 2014 |
|  | *RV* |  | AAAGCCAACATCCAATACGG |  |
| *qVvACT1* | *FW* | GSVIVT01026580001 | GTGCCTGCCATGTATGTTGCCATTCAG | Conde et al., 2015 |
|  | *RV* |  | GCAAGGTCAAGACGAAGGATAGCATGG |  |
| *qVvGAPDH* | *FW* | GSVIVT00009717001 | CACGGTCAGTGGAAGCATCA | Conde et al., 2015 |
|  | *RV* |  | CCTTGTCAGTGAACACACCAG |  |
| *qVvSUC11* | *FW* | GSVIVT01009254001 | TGTGCCAATCTCAAGTCTGCC | Pastenes et al., 2014 |
|  | *RV* |  | CCTGGGCTGCTGTTATGCTT |  |
| *qVvSUC12* | *FW* | GSVIVT01020031001 | ACCAGCCTCACCATTTATCAGAC | Pastenes et al., 2014 |
|  | *RV* |  | ATTTCATAACTGCTCTCAGGGTTG |  |
| *qVvSUC27* | *FW* | GSVIVT01034881001 | TGCTTGGCACTGACGGTACT | Pastenes et al., 2014 |
|  | *RV* |  | GCTGTAGGTGATCGCAAGAGG |  |
| *qVvHT3* | *FW* | GSVIVT01001036001 | TAATCGAACGGGGATCAAG | Hayes et al., 2007 |
|  | *RV* |  | CCCCCAGAAATCAATAAAACTC |  |
| *qVvTMT1* | *FW* | GSVIVT01013414001 | GTTGCCGTCAACTTCGCAAC | Hayes et al., 2007 |
|  | *RV* |  | GAAGGAATTTAGCTATGGCAGAG |  |

Supplementary table 2 – Cis-acting elements identified in the *VvSWEET7* promoter sequence via PLACE (Higo et al., 1999) and Plant Pan 3.0 (Chow et al., 2019). Cis-element name, sequence motifs, signaling pathway and number of copies on the promoter found on each platform are shown.

| ***Cis*-acting element** | **Sequence** | **Response** | **Number of copies** |
| --- | --- | --- | --- |
| WRKY71OS | TGAC | Pathogen defense; gibberellin | 11/10 |
| GT1GMSCAM4 | GAAAAA | Pathogen defense; salt stress | 5/5 |
| WBOXATNPR1 | TTGAC | Pathogen defense | 2/57 |
| WBOXHVISO1 | TGACT | Sugar responsive | 5/52 |
| SREATMSD | TTATCC | Sugar repression | 2/2 |
| SURE2STPAT21 | AATACTAAT | Sucrose responsive | 1/1 |
| MYBGAHV | TAACAAA | Gibberellin; sugar repression | 2/0 |
| TATCCAOSAMY | TATCCA | Sugar; Hormone regulation | 2/2 |
| GAREAT | TAACAAR | Hormone regulation | 2/8 |
| MYCCONSENSUSAT | CANNTG | Drought; cold; ABA | 18/12 |
| MYBCORE | CNGTTR | Drought | 7/25 |
| OSE2ROOTNODULE | CTCTT | Nodule; root | 9/9 |

Supplementary table 3 – Cis-acting elements identified in the *VvSWEET15* promoter sequence via PLACE (Higo et al., 1999) and Plant Pan 3.0 (Chow et al., 2019). Cis-element name, sequence motifs, signaling pathway and number of copies on the promoter found on each platform are shown.

| **Cis-acting element** | **Sequence** | **Response** | **Number of copies** |
| --- | --- | --- | --- |
| WRKY71OS | TGAC | Pathogen defense; gibberellin | 11/9 |
| GT1GMSCAM4 | GAAAAA | Pathogen defense; salt stress | 4/4 |
| WBOXATNPR1 | TTGAC | Pathogen defense | 2/63 |
| SEBFCONSSTPR10A | YTGTCWC | Pathogenesis-related | 2/2 |
| MYBGAHV | TAACAAA | Gibberellin; sugar repression | 2/1 |
| SBOXATRBCS | CACCTCCA | Sugar responsive; ABA | 1/1 |
| PYRIMIDINEBOXOSRAMY1A | CCTTTT | Gibberellin; sugar repression | 3/3 |
| CATATGGMSAUR | CATATG | Auxin | 2/6 |
| MYCCONSENSUSAT | CANNTG | Drought, cold, ABA | 20/14 |
| MYBCORE | CNGTTR | Drought | 4/37 |
| OSE2ROOTNODULE | CTCTT | Nodule; root | 5/5 |

Supplementary table 4 – *Cis*-acting elements identified in the promoter region of *VvSWEETs* expressed in the grape berry, via PLACE (Higo et al, 1999) and Plant Pan 3.0 (Chow et al., 2019). Sequence motifs, signaling pathway and number of copies on the promoter found on each platform are shown.

| **Sequence** | **Response** | **Number of copies** | | | | | | | | | |
| --- | --- | --- | --- | --- | --- | --- | --- | --- | --- | --- | --- |
|  |  | *SW1* | *SW2a* | *SW2b* | *SW4* | ***SW7*** | *SW10* | *SW11* | ***SW15*** | *SW17a* | *SW17d* |
| CATATG | Auxin | 0/6 | 0/7 | 0/5 | 0/9 | 0/6 | 4/9 | 2/5 | 2/6 | 2/6 | 4/10 |
| TAACAAR | Hormone regulation | 2/6 | 4/8 | 1/2 | 3/12 | 2/8 | 0/8 | 2/7 | 0/4 | 0/2 | 4/8 |
| GAAAAA | **Pathogen defense: salt stress** | 15/14 | 10/10 | 17/17 | 6/6 | 5/5 | 6/6 | 3/3 | 4/4 | 7/7 | 4/4 |
| CNGTTR | Drought | 1/13 | 1/22 | 4/32 | 1/16 | 7/25 | 6/37 | 4/36 | 4/37 | 7/45 | 1/24 |
| TAACAAA | Gibberellin; sugar repression | 1/0 | 3/0 | 0/0 | 3/0 | 2/0 | 0/0 | 2/0 | 2/1 | 0/0 | 4/0 |
| CANNTG | Drought; cold; ABA | 2/1 | 4/2 | 20/15 | 8/5 | 18/12 | 26/0 | 30/23 | 20/14 | 28/25 | 20/18 |
| CTCTT | Nodule; root | 8/8 | 6/6 | 6/6 | 4/4 | 9/9 | 2/1 | 7/7 | 5/5 | 5/5 | 2/2 |
| CCTTTT | Gibberellin; sugar repression | 1/1 | 2/2 | 11/11 | 2/2 | 0/1 | 3/3 | 2/2 | 3/3 | 4/4 | 4/4 |
| CACCTCCA | Sugar responsive; ABA | 0/1 | 1/1 | 0/0 | 0/0 | **1/1** | 0/1 | 0/0 | **1/1** | 0/1 | **1/2** |
| YTGTCWC | **Pathogenesis-related** | 1/1 | 1/1 | 0/0 | 0/0 | 0/0 | 0/0 | 3/3 | **2/2** | 2/2 | 2/2 |
| TTATCC | **Sugar repression** | 1/1 | 1/1 | 3/3 | 6/6 | **2/2** | 3/3 | 0/0 | 0/0 | 0/0 | 4/4 |
| AATACTAAT | **Sucrose responsive** | 0/0 | 1/1 | 0/0 | 0/0 | 1/1 | 0/0 | 0/0 | 0/0 | 0/0 | 0/0 |
| TATCCA | Sugar; hormone regulation | 0/0 | 0/0 | 1/1 | 5/5 | **2/2** | 1/1 | 1/1 | 0/0 | 2/2 | 1/1 |
| TTGAC | Pathogen defense | 2/63 | 3/59 | 1/67 | 1/54 | 2/57 | 1/63 | 6/92 | **2/63** | 3/65 | 1/79 |
| TGACT | **Sugar responsive** | 5/76 | 3/51 | 4/67 | 2/61 | **5/52** | 1/45 | 5/51 | 0/79 | 3/66 | 6/55 |
| TGAC | Pathogen defense; gibberellin | 7/0 | 7/0 | 11/0 | 5/0 | 11/0 | 10/0 | 14/0 | 11/0 | 14/0 | 14/0 |


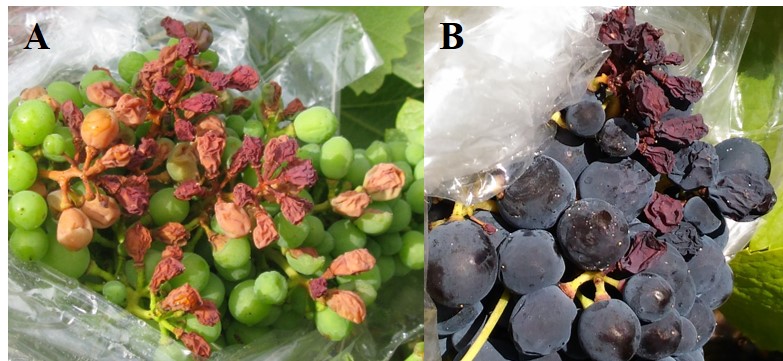
Supplementary figure 1 – Clusters of Trincadeira grapes infected with *B. cinerea* at EL32 (A) and EL38 (B), showing similar infection levels.


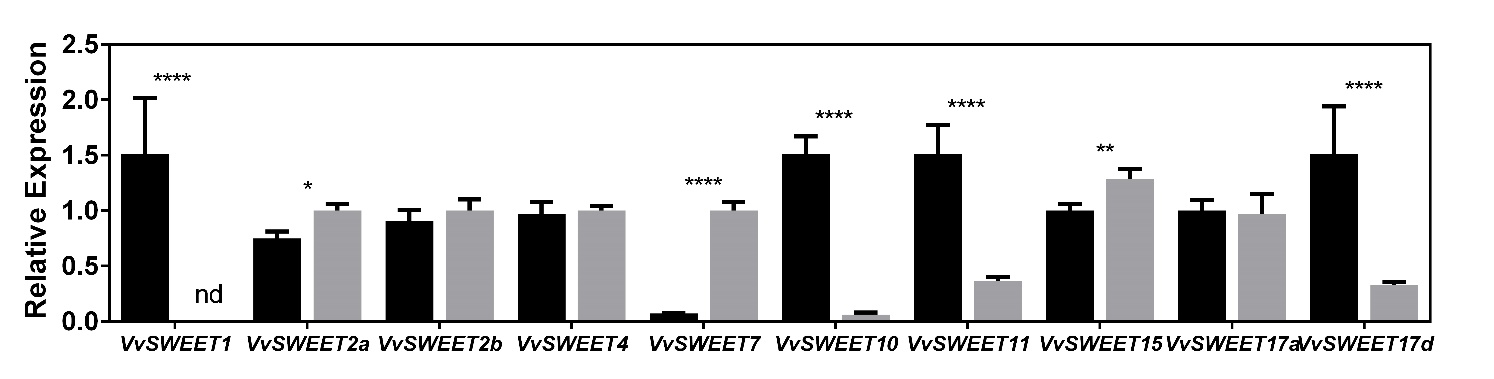


Supplementary figure 2 - Expression profile of several grapevine SWEET transporters, performed by real-time PCR in CSB suspension cultured cell, elicited 48 hours with Botrytis mycelia (■), or control ones (■). Relative expression for each gene was calculated by the Bio-Rad® CFX Manager 2.0 Software and was determined against the sample with the lowest expression level, which was set to 1. For each of the three biological replicates, after RNA extractions and cDNA synthesis, an independent qPCR analysis was performed with three internal technical replicates. Values are the mean ± SD. Asterisks indicate statistical significance (Student’s t-test: *P<0.05; **P<0.01; ***P<0.001; **** P < 0.0001).
